# Medical Informatics Platform (MIP): A Pilot Study Across Clinical Italian Cohorts

**DOI:** 10.3389/fneur.2020.01021

**Published:** 2020-09-23

**Authors:** Alberto Redolfi, Silvia De Francesco, Fulvia Palesi, Samantha Galluzzi, Cristina Muscio, Gloria Castellazzi, Pietro Tiraboschi, Giovanni Savini, Anna Nigri, Gabriella Bottini, Maria Grazia Bruzzone, Matteo Cotta Ramusino, Stefania Ferraro, Claudia A. M. Gandini Wheeler-Kingshott, Fabrizio Tagliavini, Giovanni B. Frisoni, Philippe Ryvlin, Jean-François Demonet, Ferath Kherif, Stefano F. Cappa, Egidio D'Angelo

**Affiliations:** ^1^Laboratory of Neuroinformatics, IRCCS Istituto Centro San Giovanni di Dio Fatebenefratelli, Brescia, Italy; ^2^Laboratory of Alzheimer's Neuroimaging and Epidemiology - LANE, IRCCS Istituto Centro San Giovanni di Dio Fatebenefratelli, Brescia, Italy; ^3^Department of Brain and Behavioral Sciences, University of Pavia, Pavia, Italy; ^4^IRCCS Mondino Foundation, Pavia, Italy; ^5^Division of Neurology V/Neuropathology, Fondazione IRCCS Istituto Neurologico Carlo Besta, Milan, Italy; ^6^NMR Research Unit, Queen Square MS Center, Department of Neuroinflammation, UCL Institute of Neurology, London, United Kingdom; ^7^Department of Computer, Electrical and Biomedical Engineering, University of Pavia, Pavia, Italy; ^8^Department of Neuroradiology, Fondazione IRCCS Istituto Neurologico Carlo Besta, Milan, Italy; ^9^Neuropsychology Center, ASST Grande Ospedale Metropolitano Niguarda, Milan, Italy; ^10^Memory Clinic and LANVIE - Laboratory of Neuroimaging of Aging, University Hospitals and University of Geneva, Geneva, Switzerland; ^11^Department of Clinical Neurosciences, Leenaards Memory Center, Center Hospitalier Universitaire Vaudois and University of Lausanne, Lausanne, Switzerland; ^12^University School of Advanced Studies, Pavia, Italy

**Keywords:** Alzheimer's Dementia (AD), biomarkers, diagnostic confidence, Medical Informatics Platform (MIP), disease signature

## Abstract

**Introduction:** With the shift of research focus to personalized medicine in Alzheimer's Dementia (AD), there is an urgent need for tools that are capable of quantifying a patient's risk using diagnostic biomarkers. The Medical Informatics Platform (MIP) is a distributed e-infrastructure federating large amounts of data coupled with machine-learning (ML) algorithms and statistical models to define the biological signature of the disease. The present study assessed (i) the accuracy of two ML algorithms, i.e., supervised Gradient Boosting (GB) and semi-unsupervised 3C strategy (Categorize, Cluster, Classify—CCC) implemented in the MIP and (ii) their contribution over the standard diagnostic workup.

**Methods:** We examined individuals coming from the MIP installed across 3 Italian memory clinics, including subjects with Normal Cognition (CN, *n* = 432), Mild Cognitive Impairment (MCI, *n* = 456), and AD (*n* = 451). The GB classifier was applied to best discriminate the three diagnostic classes in 1,339 subjects, and the CCC strategy was used to refine the classical disease categories. Four dementia experts provided their diagnostic confidence (DC) of MCI conversion on an independent cohort of 38 patients. DC was based on clinical, neuropsychological, CSF, and structural MRI information and again with addition of the outcome from the MIP tools.

**Results:** The GB algorithm provided a classification accuracy of 85% in a nested 10-fold cross-validation for CN vs. MCI vs. AD discrimination. Accuracy increased to 95% in the holdout validation, with the omission of each Italian clinical cohort out in turn. CCC identified five homogeneous clusters of subjects and 36 biomarkers that represented the disease fingerprint. In the DC assessment, CCC defined six clusters in the MCI population used to train the algorithm and 29 biomarkers to improve patients staging. GB and CCC showed a significant impact, evaluated as +5.99% of increment on physicians' DC. The influence of MIP on DC was rated from “slight” to “significant” in 80% of the cases.

**Discussion:** GB provided fair results in classification of CN, MCI, and AD. CCC identified homogeneous and promising classes of subjects via its semi-unsupervised approach. We measured the effect of the MIP on the physician's DC. Our results pave the way for the establishment of a new paradigm for ML discrimination of patients who will or will not convert to AD, a clinical priority for neurology.

## Introduction

The International Working Group (IWG) criteria (1, 2) define Alzheimer's Dementia (AD) as a construct based on the combination of brain amyloidosis and tauopathy (3–6). However, physicians in real-world practice primarily rely on the patient's clinical picture. Neurological diseases are classified into internationally recognized catalogs (ICDM, DSM-V) that are essentially based on phenotyping backed up by ancillary investigations.

Neuroscience seems locked in a symptom-based diagnostic paradigm moving forward without a wide-ranging biological classification scheme for brain diseases (7). To reach a complete understanding of the brain, neuroscientists cannot just focus on one level of brain organization (8), regardless of how well it may be determined at that specific level (9). An integrated, holistic, multimodal, and multilevel analysis is required. This approach could optimize the effectiveness of disease prevention and treatment and minimize side effects by considering the specific makeup of genetic information, *in vivo* biomarkers (e.g., biological or imaging-derived), and phenotypic characterization to move toward the so-called personalized medicine.

However, if neuroscience desires to understand the brain across all scales and levels of organization, it needs robust and efficient computational e-infrastructures to work together (10, 11). Computational power is mandatory to solve complex brain simulations and forecasts as quickly as possible. To cope with this need, many platforms were developed in Europe and North America in the last decade to fill the gap between data collection and information extraction (12). Neuroscience e-infrastructures, such as Laboratory Of Neuro Imaging (LONI) (13), neuGRID (14–16), C-Brain (17), and EMIF (18, 19), offer access to large databases, neuroimaging algorithms, extended computational resources, and statistical tools. More recently, the Human Brain Project (HBP), funded in 2012 by the European Commission to build an open e-infrastructure, started to provide supervised/unsupervised machine learning (ML) tools for neuroinformatics, brain simulation, neuromorphic computing, and medical informatics. Within this overarching project, the Medical Informatic Platform (MIP) was developed to allow hospitals and research centers to share medical data (20). Notably, patient records in European hospitals represent an enormous source of data (21) that are waiting to be processed using data mining algorithms and mathematical models to extract meaningful and potentially hidden information. Therefore, this vast amount of available data and emerging powerful algorithms sets the stage for a paradigm shift from a pure-hypothesis-driven medicine, as adopted currently, to a data-led objective classification strategy. This shift is the overarching aim of the MIP to find a way to combine ML tools with big data repositories already collected in hospitals to search for disease signatures (22).

The Italian Network of Neuroscience and Neurorehabilitation (RIN) (23) was founded in 2017 by the Italian Ministry of Health, and it represents the ideal starting point to test the MIP platform and validate the “disease signature” concept against clinical practice. The RIN initiative (i) encourages collaboration among the Italian Research Hospitals (IRCCS), (ii) facilitates the spread of information on clinical/scientific activities, and (iii) promotes collaborative actions at international level. Clinical, instrumental, and molecular characterization of patients represent the basis of RIN studies to identify advanced early diagnostic biomarkers, therapeutic targets, and innovative intervention strategies. Two IRCCS (IRCCS Fatebenefratelli and IRCCS Besta) of the RIN network and another external Italian hospital (Niguarda Ca' Granda) in association with the NeuroImaging lab of the Center for Health Technologies (CHT—University of Pavia) configured MIP platforms locally and participated in this study.

Data availability is as important as data comparability. Recent advances in computer science and the widespread application of big data mining have demonstrated that meaningful insights may be obtained from heterogeneous, noisy, non-standardized data (24). The variety of normal ranges is generally handled with normalization and z-scoring (25). Dichotomization is used to define abnormality when validated cut-points for abnormal scores were developed. “Messy” data may be cleaned up via smoothing, which is a technique long used in brain imaging with excellent effect (26). Missing data may be imputed using specialized interpolations, also used effectively in the averaging of brain images (27). Scaling approaches are efficiently used in case of systematic differences between cohorts for scores on the same variable (28). Strategies for coping with non-standardized data are already in place, and MIP takes advantage of all of these strategies to gather large numbers.

This study measured the performance of a supervised MIP Gradient Boosting (GB) classifier in discriminating subjects in one of the three broad categories originally attributed by clinical experts: Cognitive Normal (CN), Mild Cognitive Impairment (MCI), and Alzheimer's Dementia (AD). The initial diagnosis of the subjects was based on clinical and neuropsychological testing (6, 29, 30), because the clinical judgment is still the gold standard for the syndromic diagnosis (31). Then, we introduced the idea of classifying subjects into more than three diagnostic classes using a semi-unsupervised approach that exploited the so-called 3C strategy (Categorization, Clustering, Classification—CCC) for refinement of the common disease diagnoses, beyond what the eye can see (32). Both tools were chosen for their high degree of maturity in the MIP development factory.

Further, we investigated the perception of the clinical utility of the MIP by interviewing four clinical experts at the three participating leading Italian dementia centers to provide an assessment of diagnostic confidence (DC). We then compared the clinicians' DC values based on the MIP information with those of traditional workup information in patients with MCI.

Clinical DC truly determines patient management, and a quantifiable measure of how the ML tools of the MIP were perceived by the clinicians and how they could potentially be incorporated in a clinical context would be highly beneficial.

E-infrastructures, which may undergo substantial reshaping in the near future, involving ML algorithms and analyses of big data, may offer a solution to the aforementioned disconnection between the biological and clinical levels of disease description in the long run. If the MIP paradigm is successful, it will provide opportunities to design revolutionary *in silico* experiments to examine and elucidate the mechanisms of brain diseases in ways that were impossible until a few years ago.

## Materials and Methods

### Study Design

Our study was structured in two main parts: (1) a “group analysis” to assess the performances of two MIP ML algorithms to classify subjects in the dementia spectrum and to identify informative disease signatures and (2) a “single-case analysis” to measure the clinicians' perceived impact of the MIP tools and test the change in their DC to identify MCI that could convert to AD within 2 years. The same algorithms were used in both phases, i.e., GB (33) and CCC (34). Supplementary Figure 1 shows the workflows of the study.

### Medical Informatics Platforms

We installed and configured MIPs in three leading Italian dementia centers. The MIP platform is an e-infrastructure for data federation and big-data analysis. It was primarily developed by Center Hospitalier Universitaire Vaudois (CHUV) in the context of the HBP (https://www.humanbrainproject.eu/).

MIP enables access and analyses of anonymized medical data that are currently locked in hospitals without moving the raw data from the servers where they reside or infringe on patient privacy. End-users cannot explore the local database of each hospital. Only aggregated results or features are shared outside of the hospital.

The MIP is organized into two main parts (see Supplementary Figure 2). The MIP-Local, where only the data coordinator and associated staff can access the pseudonymized data set, and the MIP Federated, where different hospitals are connected and the end user can query and run analyses on the federated fully anonymized data sets (35). The platform provides algorithms for advanced statistical analyses, feature extraction, and predictive models via data mining and ML tools. The MIP infrastructure overall is instrumental to the identification of the biological changes associated with AD and opens new possibilities for early diagnosis by discovering otherwise unseen disease signatures.

### Data

For the “group analysis,” we used data from five different cohorts. Two of the cohorts were international research data sets, such as: The European DTI Study on Dementia (EDSD) (36) and the Alzheimer's Disease Neuroimaging Initiative (ADNI) (37) that are constituent parts of each MIP platform. The other three cohorts were Italian clinical cohorts collected from IRCCS Fatebenefratelli (FBF), IRCCS Carlo Besta (Besta), and Niguarda hospital (CHT-Niguarda). The local medical ethics committee approved each study. Participants provided written informed consent at the time of inclusion in the study for the sharing of cognitive data, fluid samples, and MRI scans. The total number of subjects originally collected was 2105 (559 AD, 847 MCI, and 699 CN). Inclusion and exclusion criteria are reported in Supplementary Table 1. The sample size was reduced to 1339 (451 AD, 456 MCI, 432 CN) to obtain data-set matching and prevent class imbalance and after visual quality control of the available T13D scans and the relative imaging biomarker segmentations of experienced researchers (AR, SD).

For the “single-case analysis,” we considered 198 MCI subjects from ADNI (118 MCI stable and 80 MCI converters to AD) with a 24-month clinical follow up as a training set. The independent subjects' data for the clinical testing came from the FBB-HUG-2014 Piramal study (38) (alias, testing set) led by the University of Geneva, with 38 MCI possibly due to AD subjects (6).

The characteristics of all the data cohorts used in the present study are reported in Table 1. Any variable with more than 40% of missing data was discarded. All the cohorts considered in the present study exposed 144 variables and included metrics in the following categories: socio-demographical, neuropsychological (including tests for verbal memory, attention, and language), volumetric (from MRI), genetic, and cerebrospinal fluid (CSF) information. In particular, Mini-Mental State Examination (MMSE) scores were available for all of the subjects considered. Surrogate imaging biomarkers were extracted from 3D T1-weighted (3DT1) MRIs. Imaging was acquired according to local Magnetization Prepared Rapid Acquisition Gradient Echo (MPRAGE) or Inversion Recovery SPoilt Gradient echo (IRSPGR) acquisition protocols. Volumetric scans were processed using the Neuromorphometrics pipeline (Neuromorphometrics, Inc., Somerville, MA) (39, 40) integrated in the MIP environment and the volumes normalized to the total intracranial volume (TIV) computed with Statistical Parametric Mapping (SPM12) considering a reference intracranial volume of 1,409 ml (41). APOE genotyping data from local genetic analyses were available for 65% of the selected individuals. CSF biomarkers were obtained using different assays across different cohorts, i.e., the Multiplex xMAP Luminex platform with Innogenetics immunoassay kit–based reagents (42) for ADNI subjects and Enzyme-Linked Immunosorbent Assay (ELISA) (43, 44) for subjects from all other cohorts, which led to different CSF biomarker distributions. To tackle this issue, biomarkers from clinical cohorts, ADNI, and EDSD were Z-scored based on the normative data specific for each cohort. All of the variables considered in the MIP were adjusted by age. A linear detrending algorithm based on age-related changes in the CN group was adopted (45) as age correction method. The adopted approach fitted a generalized linear model (GLM) for each variable and age, and the age-related changes were modeled as linear drift in the CN group only. The regression coefficient of the resulting GLM model was used to remove the age-related changes from all individuals to obtain corrected values. The assumption for the age correction method is that the age-related changes in the CN group are due to aging while the age-related changes in the MCI and AD groups includes disease-related changes.

**Table 1:** Characteristics of the data sets.

**Data Set**	**Types and availability**	**Description**	**Categories**
ADNI—Alzheimer's Disease Neuroimaging Initiative	Research data set available on every MIP web-server interface (https://services.humanbrainproject.eu/oidc/login)	A longitudinal multicenter study designed to develop clinical, imaging, genetic, and biochemical biomarkers for the early detection and tracking of AD. ADNI was originally launched in 2003 as a public–private partnership; its primary goal has been to test whether MRI, PET, biological markers, and clinical and neuropsychological assessments can be combined to measure the progression of MCI and Alzheimer's disease. It has targeted participants with AD (*n* = 222), MCI (*n* = 576), and CN (*n* = 268).	CN MCI AD
EDSD—European DTI Study on Dementia	Research data set available on every MIP web-server interface (https://services.humanbrainproject.eu/oidc/login)	A framework of nine European centers: Amsterdam (Netherlands), Brescia (Italy), Dublin (Ireland), Frankfurt (Germany), Freiburg (Germany), Milano (Italy), Mainz (Germany), Munich (Germany), and Rostock (Germany). It is a cross-sectional multicenter study characterized by 474 volumetric MRI T1-weighted scans with socio-demographic, clinical, genetic, and biological variables.	CN MCI AD
FBF	Clinical data set collected by the IRCCS Fatebenefratelli and available via MIP-Local and MIP-Federated	A cross-sectional collection including data from 1,784 patients enrolled in Brescia (Italy) and nearby areas. The data set contains socio-demographic, clinical, genotype, bio-specimen information, and MRI T1-weighted images	CN MCI AD Other
Besta	Clinical data set collected by the IRCCS Carlo Besta hospital and available via MIP-Local	A cross-sectional collection of 105 patients clinically characterized using standard neuropsychological evaluation. Volumetric MRI scans at 3T are available for all patients. CSF and DNA were collected in most cases to investigate biomarkers (e.g., T-TAU, P-TAU, AB1-42) and genotyping.	CN MCI AD Other
CHT Niguarda	Clinical data set collected by the Niguarda hospital and available via MIP-Local	A cross-sectional collection of 171 subjects. Most of subjects underwent clinical/neuropsychological assessments and volumetric MRI acquisitions.	CN MCI AD Other
Piramal	Independent external research data set not federated with the MIP infrastructure	An international, multicenter, phase 4 study where a fraction of the total patients enrolled (*n* = 71) were characterized using clinical, neuropsychological assessments, MRI (consistent with ADNI acquisition protocol), amyloid-PET, and CSF biomarkers.	MCI

*CSF, Cerebrospinal Fluid; AB1-42, amyloid beta; T-TAU, total Tau; P-TAU, phosphorylated Tau; MRI, Magnetic Resonance Imaging; CN, Cognitive Normal; MCI, Mild Cognitive Impairment; AD, Alzheimer's Disease; Other, atypical form of dementia in the clinical data sets of each hospital - these subjects were excluded from our study*.

### Categorization of the MIP Variables

Categorization in three main sets was performed to give a logical organization of the 144 variables specified in the input to the MIP supervised and semi-unsupervised algorithms.

The first category included disease diagnosis (as assigned in the original cohort) and was used in the evaluation of the supervised GB classification performances. The diagnosis feature had three levels: CN, MCI, and AD.

The second category was represented by 12 validated MRI biomarkers that very well-described the brain neurodegeneration (6). These markers were the volumes of the following structures: left/right hippocampus, left/right amygdalae, left/right anterior cingulate gyrus, left/right middle cingulate gyrus, left/right posterior cingulate gyrus, and 3rd and 4th ventricles. Both GB and CCC used this set. Furthermore, the second category was used in the CCC algorithm to define homogeneous clusters of subjects.

The third category included all the other measurements among those of the MIP we used (for a complete list of the categorization refers to Supplementary Table 2) for which we knew had, or potentially could have, a predictive value for disease risks or severity. A total of 131 markers were in this category, such as sociodemographic variables, MMSE, CSF proteins, APOE ε4 genotyping, and all other less common Neuromorphometric derived imaging variables. GB and CCC used this category. The most informative features in the third category, in addition to those in the second category, were explicitly used to define the disease fingerprint of the CCC algorithm (see Supplementary Table 3).

### Gradient Boosting

GB is a popular ML technique to solve classification problems. GB produces a prediction that exploits an ensemble of weak estimators (i.e., decision trees). GBs are used extensively in neuroimaging as because they predict outcomes with high accuracy and possess the ability to model diverse and high-dimensional data (46, 47). The main advantages of using GB are its capacity to handle variables of mixed types and its inner robustness to outliers.

In the “group analysis,” we constructed a classifier to separate patients into the following three groups: CN, MCI, and AD, i.e., the values of the first category. We performed feature relevance evaluation using a tree-based approach with a nested fold cross-validation design. The nested cross-validation consists of an inner loop for model building and parameter estimation and an outer loop for model testing. Consequently, the data set was divided into two parts: a training plus validation subset and a test subset. In the inner loop, GB models were trained with varying GB hyper-parameters (e.g., learning rate, number of estimators, maximum number of features, minimum samples split, maximum depth) based on a grid-search strategy. The validation set was used to determine the GB hyper-parameters over the grid of possible values. The performance of the resulting model, with optimized GB hyper-parameters and features, was subsequently evaluated on the test set in the outer loop. For this outer loop, we used a stratified 10-fold cross-validation scheme to divide the data into 10 equally sized parts. Nine of these parts were dedicated to the training/validation set, and one part was the test set. The 10 parts were permuted in each iteration of the outer loop so that each one was used for testing once. Finally, the GB results were averaged over the 10-folds to estimate the predictive power of the proposed model on the entire data set. To further test the GB generalizability and flexibility performances, we performed many holdout validation assessments between research and clinical data sets as well as on each clinical data set.

Before the “single-case analysis” was performed on Piramal subjects, a nested 5-fold cross-validation was used to train and test the GB on the subgroup of ADNI MCI patients. Accuracy, precision, and recall metrics were computed to assess performance.

The GB algorithm, as implemented in the open-source python Scikit-learn library (version 0.22.1), was used to perform classification (48).

### CCC Algorithm

The CCC algorithm is a semi-unsupervised ML tool developed by Tel Aviv University (32, 34). CCC was used to obtain a homogeneous subjects' clustering and to identify potential combinations of biomarkers for a deep characterization of the disease.

In the first step, the algorithm considered the so-called second category variables that we defined earlier, which was composed of 12 imaging features, plus the clinical diagnosis to identify the optimal number of clusters present in the patient's data set. A Random Forest (RF) selection method ranked the weight of each feature, and the number of clusters was derived using Gap statistic (49). Then, the clustering PAM (Partitioning Around Medoids, also known as K-medoids) algorithm (50) was applied to label each subject into one cluster. To discuss the meaning of the created classes, we cross-classified the clusters generated with the original diagnosis and demographic and clinical variables. Finally, the still unused variables of the third category were exploited as potential features to define and expand the disease fingerprints of the created clusters. In particular, the CCC algorithm selected a subset among the most promising of the 131 third category variables. To do this, a feature selection process was performed via the RF mechanism. RF assigned a weight of importance to each of the third category feature (51), and the most informative were selected. In this way, CCC identified markers that were useful to define the final disease fingerprint.

Further, the CCC algorithm, prying on the selected informative third category variables and hierarchical decision trees with out-of-bag validation (52), generated a matching matrix that represented the ability to correctly classify subjects according to the K-medoids clusters previously identified.

Parallel coordinate plots were used to allow comparison of the derived fingerprints, including confidence intervals. Data were normalized by subtracting the minimum and dividing by the maximum of all observations. This method allowed comparisons of variables of different scales and keep preserved the shape of the different distributions.

The CCC is an MIP semi-unsupervised strategy and is based on the “randomForest,” “cluster,” “rpart,” “psych,” and “ggparci” R packages.

### Clinical Impact

The “single-case analysis” we performed tested and measured how physicians are influenced in their DC by the MIP information. The clinical question was to verify the perceived usefulness of the MIP to classify whether an MCI subject would convert to AD or would remain stable in the next 2 years. Physicians were also asked to express the etiological causes of the disease. The 4 physicians (SG, GB, PT, SC) had a long experience with the diagnostic use of AD biomarkers and were aware of the most recent research diagnostic criteria (1, 2, 5) for AD. To provide useful information to the clinicians, the MIP algorithms were trained on 118 MCI stable and 80 MCI converters to AD from ADNI. Thirty-eight MCI subjects from Piramal were analyzed as multiple single cases. Piramal 3DT1 scans were used to extract features using the Neuromorphometric pipeline, and all the data were post-processed with both GB and CCC, while the clinical assessments were collected using an *ad hoc* web-based questionnaire.

The physicians interviewed did not have specific constraints about the diagnostic etiologies. All etiological diagnoses were grouped ex-post into:

- Pathophysiologically AD-related: MCI due to AD
- Not pathophysiologically AD-related: MCI not due to AD, MCI due to frontotemporal dementia (FTD); MCI due to Vascular Disease (VD); MCI associated with psychiatric disorder; Suspected Non-Amyloid Pathology (SNAP); and Normal Aging (Supplementary Table 4).

The four physicians provided their initial estimate of DC. The following available information for each Piramal subject, describing the initial physicians' evaluations, was used: (1) age, sex, education, and other socio-demographic information; (2) neurological examination; (3) MMSE score; (4) neuropsychological assessment of long-term memory, executive functions, language domains expressed as both raw and equivalent scores (53); (5) MRI visual assessment performed by an expert neuro-radiologist; and (6) amyloid beta (AB1-42), total Tau (T-TAU), and phosphorylated Tau (P-TAU) CSF levels.

Then, the four physicians were asked to reassess the same subjects by taking into consideration the report from the MIP, which included the following factors: (i) the density function distributions built from the Neuromorphometrics analysis of established imaging biomarkers (i.e., hippocampus, cingulate, entorhinal, parahippocampal, and superior temporal gyrus) showing three standard deviations (Supplementary Figure 3); (ii) the CCC K-medoids clustering (**Figure 2B**); and (iii) GB classification results, which ranged from a stable stage to a very probable conversion stage (Supplementary Figure 4), depicted via a partial dependence plot (PDP). The four physicians provided their final estimate of DC on a structured scale (ranging from 0 to 100%). Clinicians finally reported whether the ML information of the MIP affected their DC assessment for each subject using a four-level Likert scale (possible answers: YES significantly; YES somewhat; YES slightly; NO not at all).

### Statistical Analyses

As far as “group analysis” is concerned, MANOVA was initially performed in R (version 3.5.1) to verify the comparability of the five data sets in the three diagnostic classes (CN, MCI, and AD).

Kruskal–Wallis and Fisher's exact *post hoc* tests (α = 0.05) were, respectively, used to test continuous or binary markers differences between the three diagnostic groups.

With regard to “single-case analysis”, Fleiss' Kappa inter-physicians reproducibility on etiological diagnosis, with and without MIP information available, was performed in R (v3.5.1) using the “dplyr” and “irr” packages. We used the python Scikit-learn library (version 0.22.1) for the “single-case analysis” to generate the PDP of GB showing each Piramal subject plotted in a 2D probability space. PDP depicted the probability of being an MCI converter using a canonical 2D space.

## Results

### Group Definition

To test the comparability, we performed a MANOVA test on the three diagnostic classes for the five data sets (see Supplementary Table 5). All data set comparisons performed were statistically not dissimilar for the same diagnostic class (*p* > 0.05). Our group included 1,339 subjects, who were stratified as follows: 432 CN (195 FBF, 28 Besta, 3 CHT-Niguarda, 100 ADNI, 106 EDSD); 456 MCI (103 FBF, 16 Besta, 18 CHT-Niguarda, 269 ADNI, 50 EDSD); 451 AD (200 FBF, 15 Besta, 135 ADNI, 101 EDSD).

Demographic and clinical information are presented in Table 2. The AD subjects were older, were less educated, and had lower MMSE compared to the other groups. We observed a female gender preponderance in the CN and AD groups. AD subjects were more often APOE ε4 carriers than MCI and CN subjects. Within the CN group, when quantified, 9% of subjects had abnormal AB1-42 CSF marker, and 82 and 97% of subjects had this abnormal marker in the MCI group and AD group, respectively.

**Table 2:** Group characteristics.

**Characteristics**	**Cognitively normal - CN; *n* = 432**	**Mild cognitive impairment - MCI; *n* = 456**	**Alzheimer disease - AD; *n* = 451**	***P***
Age—years	65.47 (9.31)	71.18 (8.08)	73.70 (7.97)	<0.001 ^¢, ¥, Ξ^
Gender—M/F	172/260	228/228	168/283	<0.001 ^¢, Ξ^
Education—year	12.60 (4.36)	13.53 (4.40)	10.64 (4.75)	<0.001 ^¢, ¥, Ξ^
MMSE—raw score	27.91 (1.27)	26.33 (2.01)	20.54 (4.25)	<0.001 ^¢, ¥, Ξ^
% APOE ε4 carriers				
–/–	80%	65%	25%	
–/+	18%	30%	64%	
+/+	2%	5%	11%	
% AB1-42 abnormal	9% [23%]	82% [68%]	97% [38%]	<0.001 ^¢, ¥, Ξ^
% T-TAU abnormal	4% [23%]	17% [69%]	90% [38%]	<0.001 ^¢, ¥, Ξ^
% P-TAU abnormal	4% [23%]	18% [69%]	62% [37%]	<0.001 ^¢, ¥, Ξ^

*Data are expressed as the mean values. Standard deviation is reported in round brackets. The overall frequencies of the CSF biomarkers in each group are reported in square brackets. AB1-42, amyloid beta; T-TAU, total Tau; P-TAU, phosphorylated Tau; n, numerosity; P, significance on Fisher's exact post hoc test or Kruskal–Wallis multiple comparison test with the Benjamini–Hochberg method (¢, Significant difference between CTR and MCI; ^¥^, Significant difference between CTR and AD; ^Ξ^, Significant difference between MCI and AD)*.

For the Piramal data set, 53% of the 38 MCI subjects were male, the mean age was 71.03 ± 7.72 years, and the mean raw MMSE score was 26.3 ± 2.14. The overall frequencies of the considered clinical measures and biomarkers assessed are reported in Table 3.

**Table 3:** Descriptive statistics of the clinical variables and biomarkers of the Piramal patients.

**Characteristics**	**Piramal mild cognitive impairment group**	
	**Biomarker frequency**	**Mean (SD)**
Age—years	100%	71.03 (7.72)
Gender—M/F		20/18
Education—year	100%	11.29 (4.63)
MMSE—raw score	100%	26.3 (2.14)
Logical Memory—EQS	100%	0.89 (1.11)
AVLT Immediate—EQS	100%	2.24 (1.42)
AVLT Delayed—EQS	100%	1.53 (1.39)
Digit Span Forward—EQS	100%	3.08 (1.12)
Digit Span Backward—EQS	53%	2.8 (1.20)
TMTA—EQS	71%	3.26 (1.40)
TMTB—EQS	71%	2.55 (1.34)
Letter Fluency—EQS	100%	3.21 (1.17)
Category Fluency—EQS	100%	2.42 (1.39)
% APOE ε4 carriers	40%	
–/–		73%
–/+		27%
+/+		0%
% AB1-42 abnormal	100%	66%
% T-TAU abnormal	100%	47%
% P-TAU abnormal	87%	11%

*EQS, Equivalent Score; SD, standard deviation; MMSE, Mini Mental State Examination; AVLT, Rey Auditory Verbal Learning Test; TMT, trail-making test; AB1-42, amyloid beta; T-TAU, total Tau; P-TAU, phosphorylated Tau*.

### Gradient Boosting Results

In the “group analysis,” the most informative features selected by the GB classifier in order of importance were AB1-42, T-TAU, P-TAU, and MMSE. Other important, albeit less informative features, selected across the groups were left hippocampus, left amygdala, and 4th ventricle. The complete rank of features is reported in Supplementary Table 6.

In the nested 10-fold cross-validation that combined all of the informative features, GB resulted in an accuracy of 85 ± 4% in classifying CN, MCI, and AD subjects. Generally, exclusion of the CSF variables led to a deterioration of the GB performance. The results of GB in holdout validations, which was used to test independently each clinical data set, resulted in overall better performances, with an accuracy that ranged from 91 to 95%. Table 4 shows the Accuracy, Precision and Recall metrics. GB revealed high precision and high recall in the “group analysis,” which related to a low false-positive and low false-negative rates, respectively.

**Table 4:** Results from GB classifiers with different validation techniques.

**Validation technique**	**Features used**	**Train set**	**Test set**	**Accuracy**	**Precision**	**Recall**
(Nested) 10-fold cross-validation	143 (SCD., NPSY., MRI, GEN., CSF)	All	All	85 ± 4%	91 ± 5%	91 ± 5%
	140 (SCD., NPSY., MRI,GEN.)	All	All	69 ± 1%	79 ± 8%	79 ± 7%
Holdout validation	143 (SCD., NPSY., MRI, GEN., CSF)	ADNI + EDSD	FBF + Besta + CHT-Niguarda	94.63%	94.32%	94.70%
	143 (SCD., NPSY., MRI, GEN., CSF)	ADNI + EDSD + Besta + CHT-Niguarda	FBF	95.18%	95.41%	95.41%
	143 (SCD., NPSY., MRI, GEN., CSF)	ADNI + EDSD + FBF + CHT-Niguarda	Besta	91.52%	91.33%	88.77%
	143 (SCD., NPSY., MRI, GEN., CSF)	ADNI + EDSD + FBF + Besta	CHT-Niguarda	95.23%	96.43%	97.22%
(Nested) 5-fold cross-validation	143 (SCD., NPSY., MRI, GEN., CSF)	ADNI (stable & conv)	ADNI (stable & conv)	62 ± 1%	66 ± 1%	70 ± 1%

*SCD, sociodemographic variables; NPSY, neuropsychological variables; GEN, genetic variables. All, ADNI + EDSD + FBF + Besta + CHT-Niguarda data sets; stable, 118 MCI stable subjects from ADNI; conv, 80 MCI converter to AD or other form of dementia from ADNI*.

In preparation of the “single-case analysis,” we assessed the GB performances for identifying MCI converters to AD using a nested 5 cross-fold validation strategy performed on 198 MCI ADNI patients. These patients were followed longitudinally for 24 months and expert ADNI neurologists performed their clinical assessments. The final assessment of ADNI physicians represented our ground truth and yielded an accuracy of 62 ± 1%. This base allowed us to fine-tune the GB hyperparameters in preparation of the multiple single-case analyses on the Piramal data set.

### Categorization, Clustering, and Classification Results

**Figure 1:**
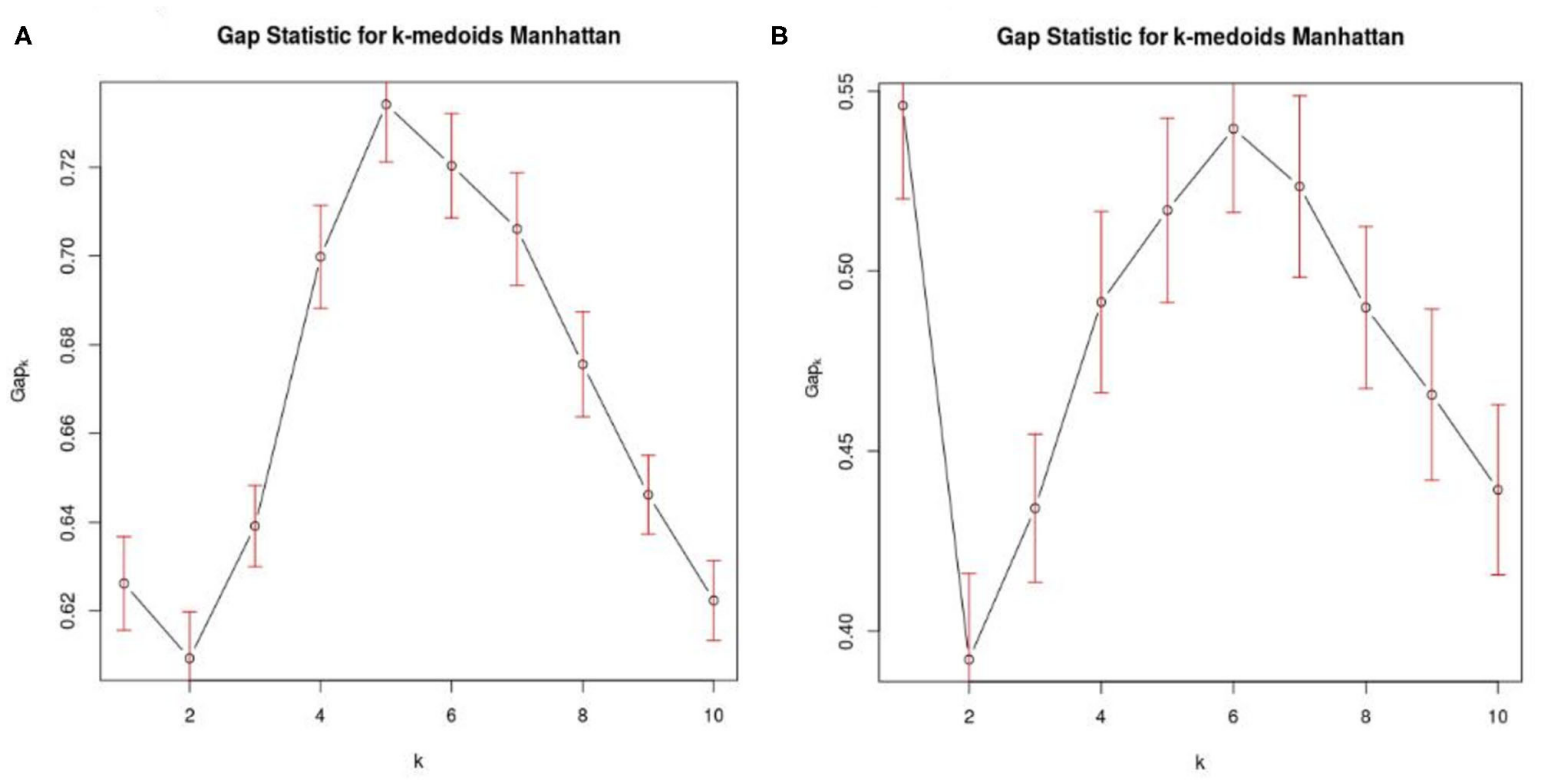
Optimal number of clusters. **(A)** Shows Gap statistics considering AD, MCI, and CN from the 5 data sets (ADNI, EDSD, FBF, Besta, CHT-Niguarda), while **(B)** represents the MCI population from ADNI that was used in the “single-case analysis.” K denotes the maximum gap, and it represents the best number of homogeneous clusters definable from our data.

**Figure 2:**
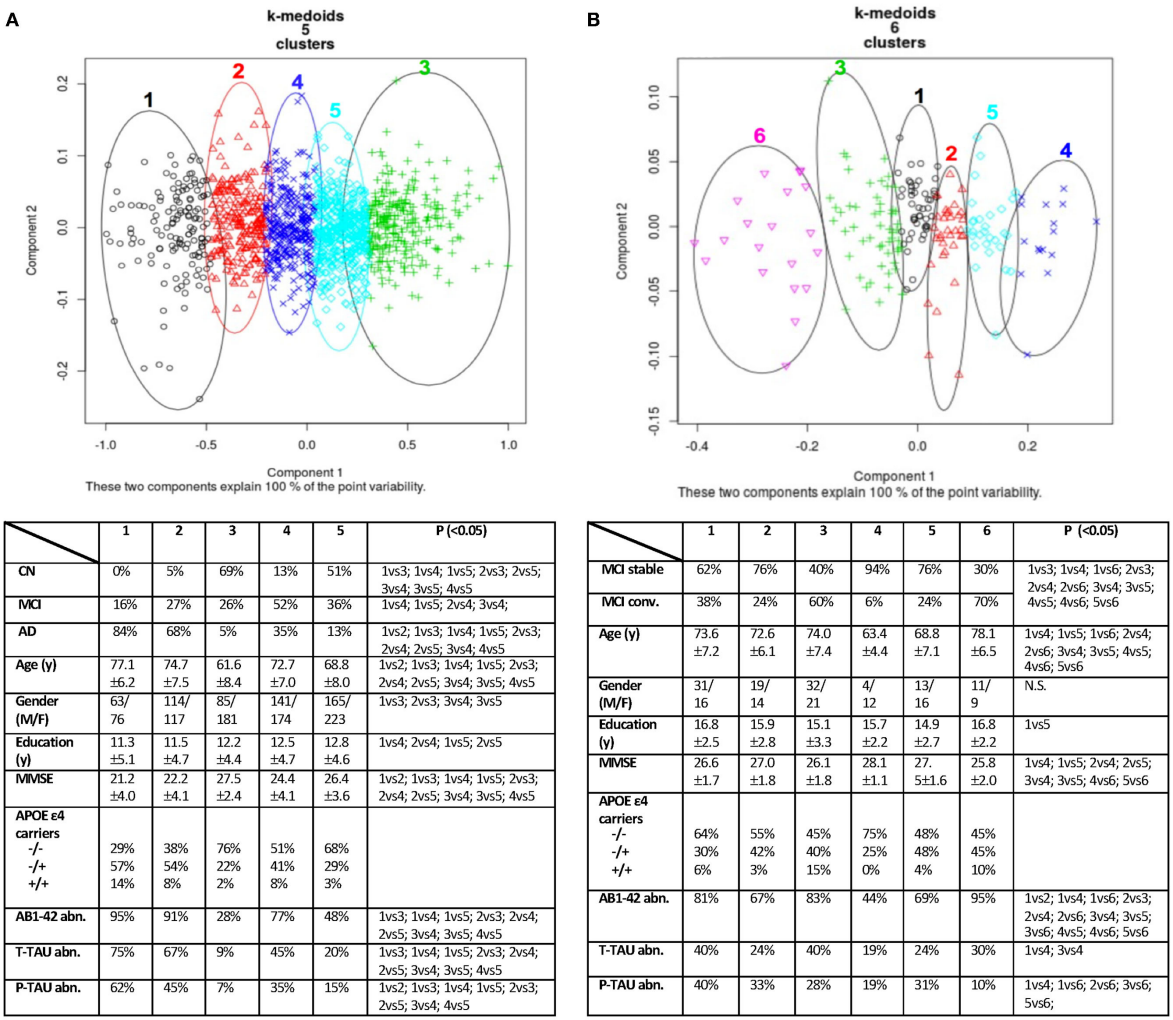
Data clustering obtained using PAM (K-medoids) algorithm. On the left **(A)**, the “group analysis” result with five clusters. On the right **(B)**, the “single-case analysis” result with six clusters. Each cluster of **(A,B)** was cross-validated in two synoptic tables against known diagnostic classes, common demographic and clinical variables. P: significance on Fisher's exact *post hoc* test or Kruskal–Wallis multiple comparison test with the Benjamini–Hochberg method; α = 0.05 level. CN, Normal Cognition; MCI, Mild Cognitive Impairment; AD, Alzheimer's Disease; y, years; MMSE, Mini Mental State Examination (raw score); AB1-42, amyloid beta; T-TAU, total Tau; P-TAU, phosphorylated Tau; abn., abnormal; N.S., not significant.

**Figure 3:**
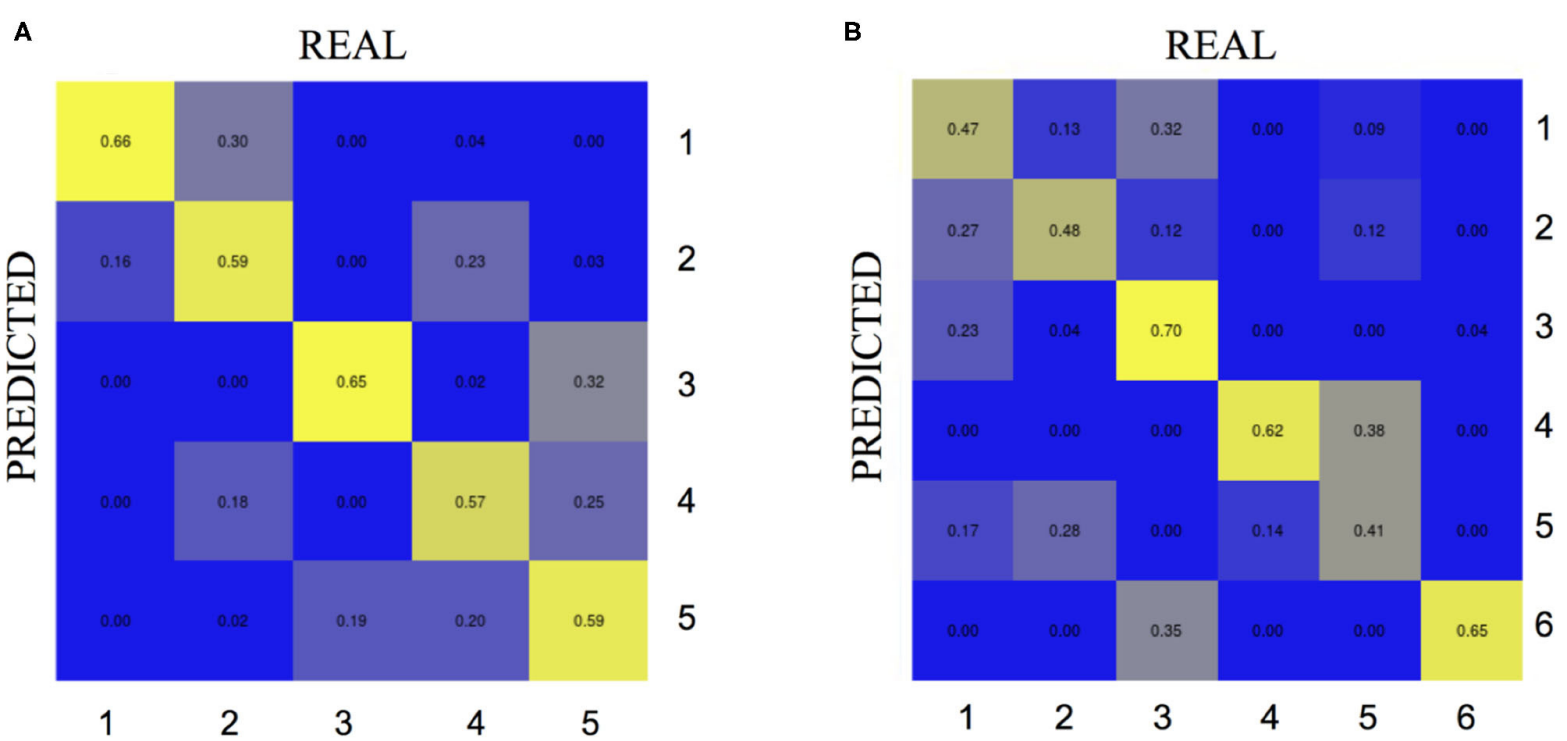
Matching matrix from the CCC algorithm considering the most important potential features. On the left **(A)** the results for the “group analysis” exploiting 24 biomarkers. On the right **(B)** the results for the “single-case analysis” exploiting 17 biomarkers. Each cell in the confusion matrix shows the percentage of predictions made for the corresponding true label. REAL, true labels of the clusters defined via PAM using first and second category variables previously defined; PREDICTED, predicted clusters via hierarchical decision trees and third category variables previously established.

**Figure 4:**
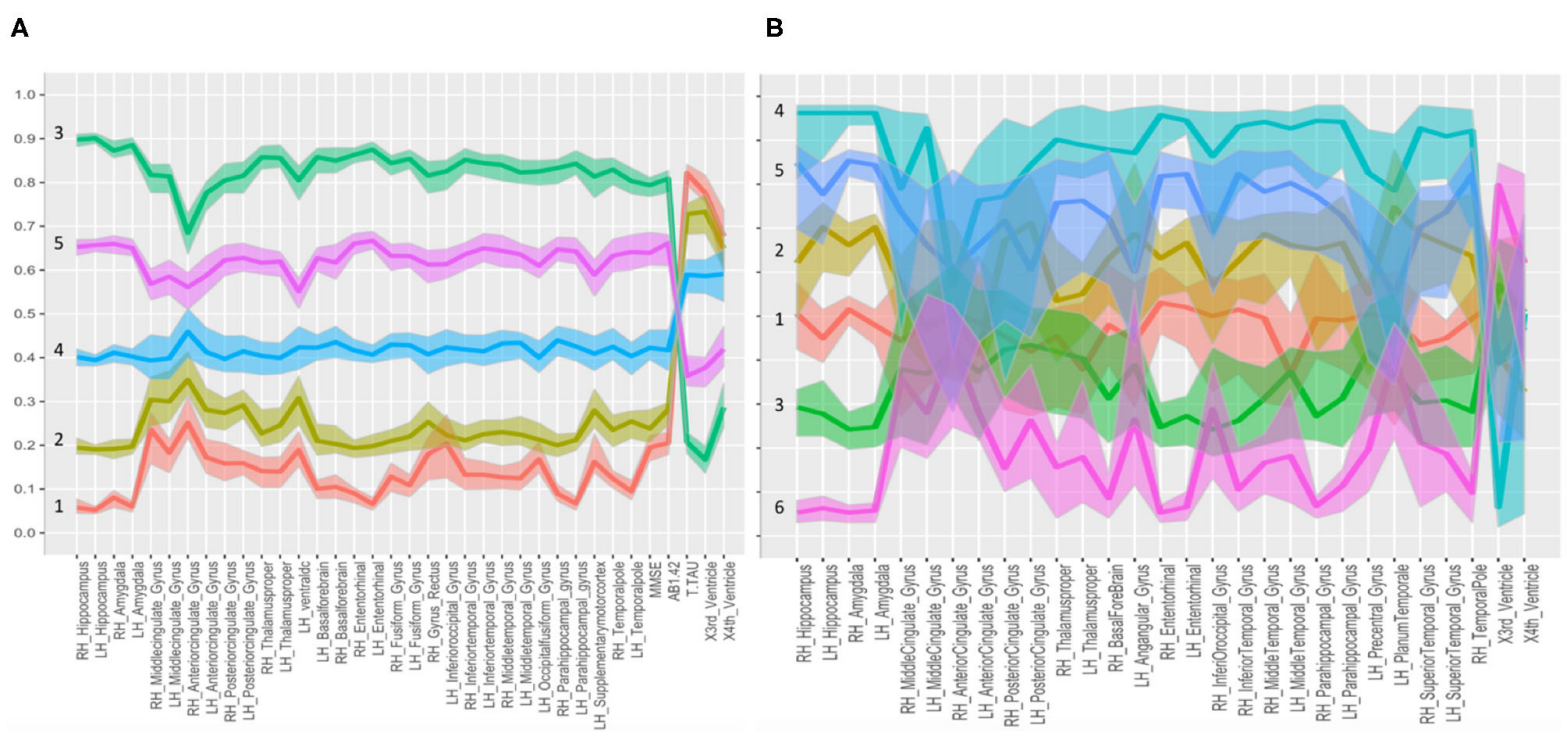
Parallel coordinate plots with confidence intervals. Distinctive plots of the different clusters identified in the “group analysis” **(A)** and “single-case analysis” **(B)** are shown. Every line connects the medians of the features identified by the CCC for each group. The variables were normalized to the [0,1] scale. In **(A)**, the five classes are well separated; in **(B)**, the confidence bands of the six MCI subclasses were partially overlaid. Both panels report the second category variables plus the informative third category variables, selected as potential features, useful to define the disease fingerprint for each cluster. Biomarkers used to graph fingerprints of **(A,B)** are reported in Supplementary Table 3.

For the “group analysis,” starting from the original diagnosis (first category) and the 12 previously defined second category markers (i.e., neurodegeneration appraised by regional atrophy), the Gap statistics identified that five homogeneous classes would be appropriate (Figure 1A). The PAM assigned each subject to one of the five clusters. For visualization purposes, the two most important components were used to draw the data points represented in Figure 2A. Noticeably, the leftmost cluster (i.e., cluster number 1) was the cluster with the maximum percentage of AD (84% AD, 16% MCI, 0% CN), the lowest MMSE score (21.2 ± 4.0), the highest prevalence of APOE4 carriers (71%), and the highest level of abnormal AB1-42, T-TAU, and P-TAU proteins in the CSF, while the rightmost cluster (i.e., cluster number 3) contained primarily CN (5% AD, 26% MCI, 69% CN) and had the highest MMSE score (27.5 ± 2.4), the lowest prevalence of APOE4 carriers (24%), and the lowest level of abnormal AB1-42, T-TAU, and P-TAU proteins in the CSF. The CCC identified 24 potential informative features, in addition to the 12 features of the second category, that defined the disease fingerprint of the five clusters. A matching matrix summarizing the results of predicting the five classes using just the 24 potential markers is reported in Figure 3A. The values on the diagonal (i.e., true positive) were higher than the others, which suggests that these potential features may be used to fairly define the definitive disease fingerprint of each patient. To understand the meaning of the five new created classes, we present a distinctive profiles plot in Figure 4A. The median line of each class is well segregated from the others, and the confidence intervals for each biomarker rarely overlapped to the others, which suggests good cluster separations and new informative purely data-driven diagnostic classes.

For the “single-case analysis,” the Gap statistics identified six subclasses (Figure 1B) from the 198 MCI (118 stable and 80 converter) patients acquired from ADNI (Figure 2B). The leftmost cluster (i.e., cluster number 6) was the cluster with the maximum percentage of MCI converters (70% converter, 30% stable), the lowest MMSE score (25.8 ± 2.0), the highest prevalence of both APOE4 carrier (55%), and AB1-42 positive (95%) subjects, while the rightmost cluster (i.e., cluster number 4) contained primarily MCI stable (6% converter, 94% stable) and had the highest MMSE score (28.1 ± 1.1), the lowest prevalence of APOE4 carriers (25%), and the lowest level of abnormal AB1-42, T-TAU, and P-TAU proteins in the CSF. The matching matrix (Figure 3B) was built considering 17 potential informative markers. The definitive disease fingerprints of the new six classes were graphed combining the 17 markers, with the 12 features of the second category, and using a parallel coordinate plot (Figure 4B).

### Clinical Utility of the ML Tools in the MIP

The information provided by the MIP tools did not modify the inter-rater agreement of the four physicians. The inter-rater concordance on the etiologic diagnosis (pathophysiologically AD related vs. not pathophysiologically AD related) was moderate, with a Fleiss' kappa of 0.521 (*p* < 0.001).

**Figure 5:**
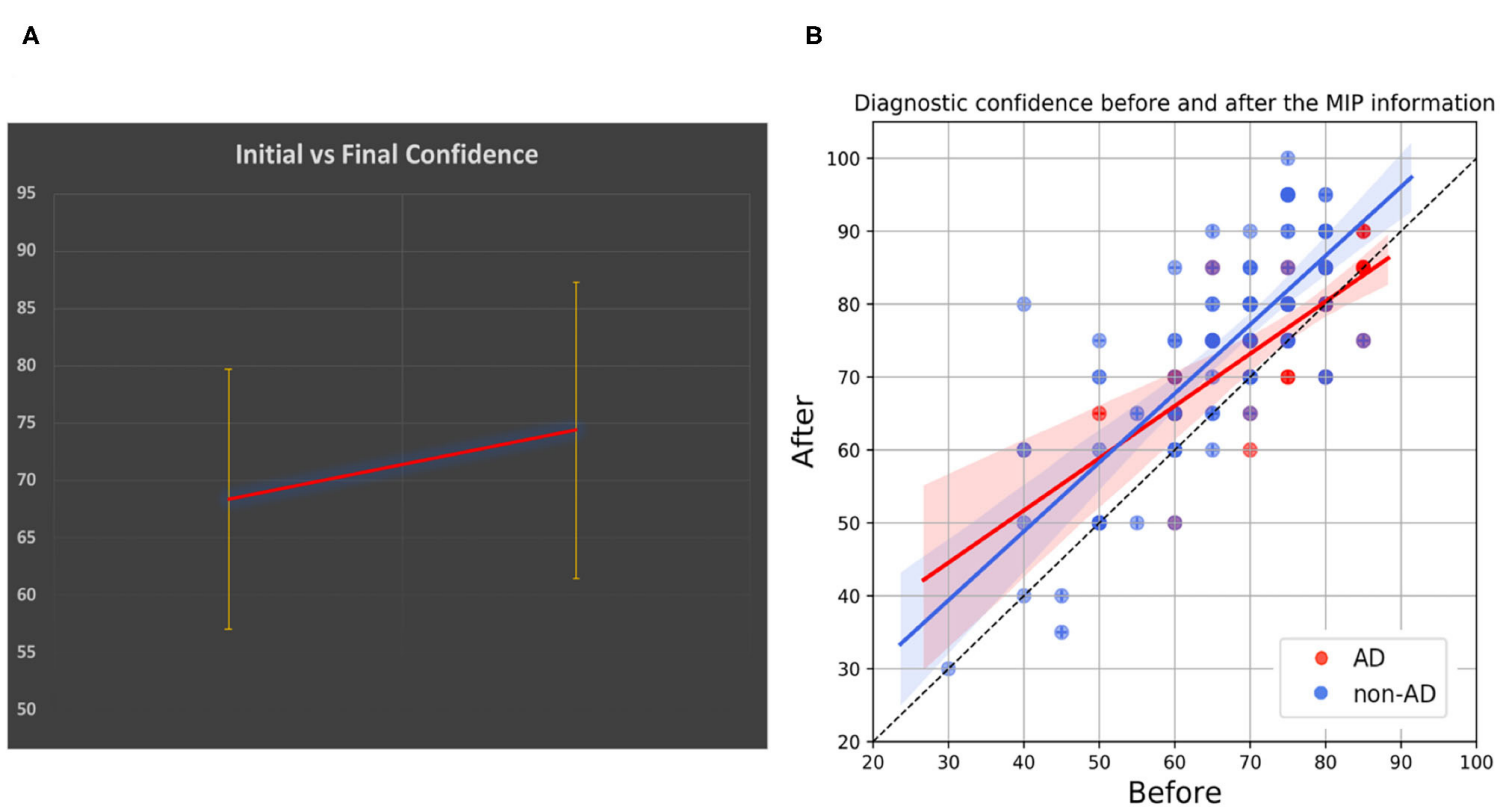
MIP and diagnostic confidence. **(A)** Shows the incremental clinicians' diagnostic confidence (DC). **(B)** Shows a scatter plot describing the DC before and after disclosure of the MIP information. Points that lay on the bisector of the plane are cases for which there was no change in DC, whereas points that are further from the bisector are cases for which the change in DC was largest. Color transparency was used for all the points to achieve the best visualization of patients having same DCs. AD, Pathophysiologically AD-related group; non-AD, Non-pathophysiologically AD related.

The MIP information induced a change in the original etiological hypothesis in three evaluations. We registered 10 changes in the hypothesis of subject conversion with a nonsignificant Fisher's exact test equal to 0.909 at alpha = 0.05 level. The registered increment in diagnostic confidence before and after the MIP information disclosure was equal to +5.99 percentage points (Figure 5A). In Figure 5B, the DC is described in a scatter plot. Points drawn in cooler colors, which denote non-pathophysiologically AD-related subjects, are in most of the cases located above the bisector of the plane, thus showing that the MIP information increased the DC of the physician's prediction. Points drawn in warmer colors (i.e., pathophysiologically AD related) are above the bisector, for lower initial DC values, and become closer to the bisector once the initial DC value increases. Therefore, the impact of the MIP was more evident when the initial DC of MCI conversion to AD is low (<70%).

When directly asked about the perceived impact of the MIP tools on the final DC, in 20% of cases clinicians reported that the additional information did not change their initial DC. In 36% and in 29% of cases, they felt “somewhat” or “slightly” impacted, respectively. In 15% of cases, they significantly changed their initial belief.

## Discussion

The present study demonstrated that the MIP provided dedicated tools for CN, MCI, and AD classification reaching in our “group analysis” similar performance to the state of the art ML algorithms (54–56). We used the supervised GB classifier based on the combination of sociodemographic, MMSE, APOE ε4 genotype, CSF, and MRI data to predict the patient's stage in the entire disease spectrum with fair accuracy. Such level of accuracy was expected and can be explained because the clinical data used for prediction partially correlated with those used for making the supervised diagnosis at baseline. GB performances were higher for the hospital holdout tasks than in cross-validation and reached a maximum peak of 95% of accuracy in two out of the three clinical cohorts. The following possible reasons explain these results: (i) the evaluation depends on which data points end up in the training set and which end up in the test set, furthermore the evaluation often may be significantly different depending on the hospital cohort subdivision; (ii) the holdout strategy is subjected to higher variance because of the smaller size of data, and the samples used might not be always representative; and (iii) the holdout method might provide only an estimate of the “true error rate” (accuracy) of the classifier.

Considering the power of the MIP to federate features from a limitless number of data sets across the globe, our results are encouraging and exciting. Indeed, the MIP platform will need to be further tested in future studies with other cohorts. To quantitatively measure the performance of the GB in discriminating MCI stable vs. converters, we used the longitudinal ADNI data set, which represented an indirect validation and a plausibility test of the MIP tool granting a 62% of accuracy. This level of performance is consistent with the results proposed in the literature (57–59) using similar features available in our study. It is realistic that GB performances were suboptimal when considering a complex classification task, such as the prediction of MCI conversion, compared to the earlier distinction between well-established diagnostic classes, i.e., CN, MCI, and AD. GB is an advanced and powerful ML tool; however, it cannot provide a real change in the diagnostic paradigm. This issue is naturally ingrained in the supervised algorithm design that cannot allow the definition of new diagnostic classes. However, GB was judged to be informative for the physicians in the “single-case analysis.”

The use of the MIP with the CCC algorithm, based on its agnostic data-mining approach called known-group validity, identified finer clusters and provided a newer data-led stratification of disease based on each patient's brain features. We presented a semi-unsupervised approach that separated patients into homogenous groups according to well-known imaging biomarkers of neurodegeneration. Notably, in the “group analysis,” we found five classes, which were identified in another recent study (60), that might represent a more careful distinction of subjects compared with the three classical broad diagnostic groups, such as CN, MCI, and AD. We enriched the conventional imaging biomarker set of the second category with 24 additional potential markers to demonstrate the consistency of the new classes. Overall, the matching matrix produced interestingly results in which many true positive predictions were located on the diagonal of the table, which gave reliability to the selected potential features of the third category to discriminate the five clusters. The matching matrix showed small prediction errors because percentage values outside the diagonal were generally small. One possible explanation for the mismatched cases is that there were some partially interrelated features among the features used for the classification. CCC defined groups that were characterized by sets of quantifiable biological and clinical variables (alias disease fingerprint) that well defined the biological makeup of the disorders.

The same approach was used in the “single-case analysis” to obtain six classes, with 17 potential features that were useful to define the disease fingerprint of the MCI subgroups in combination with the conventional imaging biomarker set of the second category. We do not claim that our findings present the best current views on the problem. Although the perceived impact of clinicians was in favor of the MIP tools, how well the CCC strategy identified the risk of conversion and the etiology must be yet determined because information about follow-ups and postmortem examinations is not provided in the Piramal database. Future studies must thus be designed to validate this stimulating finding. Moreover, expert knowledge in enlightening the data, such as diversification of questions to different cognitive domains or other biomarker measurements, could help create even more refined clusters of disease arrangement.

In the “single-case” part of this study, MIP provided an added value to clinicians' DC even when used on top of the traditional diagnostic workup. MIP in combination with neuropsychological assessments and CSF biomarkers had not a trivial impact with an increment equal to +5.99% in the final DC. This result was consolidated considering the perception of the physicians involved in the scoring of MCI subjects who declared that MIP information influenced their DC with an impact that was rated from “slight” to “significant” in 80% of the cases.

Because the MIP platform is by definition a simple, quantitative, reproducible tool that requires fast training, it may provide an important added value in the diagnostic process of the dementia.

Few discordant and contradictory MIP results were registered by physicians between CCC and GB. These were primarily due to algorithm implementation differences in terms of ML approaches and different selection mechanisms of biomarkers to be used. CCC performed a feature pruning mechanism, GB instead was more robust to overfitting and tried to maximize the pathological information using all of the available features. Agreement between MIP tools is thus subject to technical differences, which may lead to slightly different diagnostic conclusions on the same patient. Therefore, the physician is absolutely essential and not replaceable by any artificial intelligence system, not even the most advanced (61, 62). However, discordant cases may also be due to borderline results.

This said, the MIP could contribute to help physicians in their daily workup and in their patient management and it may be used to test other clinical hypotheses. One of the main advantages of adopting MIP and its integrated ML tools is to combine hundreds of pieces of information (i.e., features) of different nature in a few graphs for the benefit of clinicians, which simplifies how to process the wealth of information available.

Analyzing health and especially hospital data, which are normally much noisier than research data, requires researchers to face some challenges. Some of the major challenges are reported hereinafter. Compensatory mechanisms may obscure the linkage between biological markers and disease (63). For example, two subjects with the same brain images do not necessarily share the same clinical manifestation. It is not only due to the complexity of the disease or the inefficiency of the marker but also by the fact that different compensatory mechanisms exist from one subject to another, which generate a miscellaneous effect on the clinical phenotype. The rate of agreement between physicians is generally modest, which means that supervised ML approaches may be partially invalidated by a poor initial classification. Recent major pathological reviews reported that the diagnosis of AD was no better than 60–70% accurate (4, 5). This had generated a great debate on the potentially greater usefulness of semi-unsupervised or completely data-driven approaches. Big-data analyses may increase the possibility to tease out irrelevant biomarkers that were identified by chance in reduced populations. To tackle this methodological problem, a well-founded and validated selection process for features and patients must be performed.

The CCC approach clearly showed the presence of disease classes beyond clinical ones in the overall group of patients and within the MCI group itself. Notably, the overall patient group was divided into classes with clearly defined features that did not overlap with each other and maintained a specific order (i.e., in “group analysis,” group 3 was above all other groups for all features, and group 1 was characterized by the lowest values for all features, with the inversion of the 4th ventricle size). Therefore, the disease fingerprint we can derive for each patient is appealing and may be beneficial for their selection in the future clinical trials, as long as a deeper phenotyping of the identified clusters will be available and confirmed by the clinicians.

Unfortunately, the number of new drugs entering the market is scarce, and many big-pharma companies have recently stopped investing in this area. This situation is a direct consequence of the lack of a clear causative understanding of AD, and it is difficult to find new treatments for brain diseases in this scenario. Therapies in AD normally focus on cholinesterase inhibitors, which are only given after the onset of symptoms.

To change this perspective toward a new paradigm, the MIP platform is gathering a large number of patients and healthy controls to characterize the disease from quantifiable biomarkers instead of gathering the patients' referred symptoms and from few non-standardized instrumental assessments.

We showed how the MIP platform could provide accurate and innovative ways to detect, stratify, and classify patients. We identified possible subject-specific biological signatures that are not captured by the traditional and oversimplified diagnostic categories. These fingerprints should clarify the researchers and physicians on the causes and mechanisms of AD to highlight targets for effective personalized treatment approaches and upcoming prevention studies.

The present study was cross-sectional, and it is still far from a real big analytics experiment despite the gathering of a large number of multidomain features from different cohorts of patients. Future efforts should be made to identify sets of biomarkers that distinguish different disease trajectories longitudinally. This goal requires the integration of data sets with follow-ups within the MIP and appropriate spatiotemporal mathematical models (64–66), which are currently absent in the platform, that may be used as a source of information on biological mechanisms that drive AD progression.

Several unanswered questions must be clarified in the future, such as the following: Is the disease signature a “guarantee of success” for new drug development? Will biological fingerprint characterization predict disease manifestations more accurately? What is the phenotypic difference between close and distant disease signatures?

The present study has a few limitations. The RIN network includes participating Italian clinical centers, which may represent a selected group that is more likely to make use of innovative biomarkers for diagnosis. In principle, we cannot exclude that different ML tools, hosted within the MIP but not chosen for our specific study, could have different impacts, better or worse, on the classification performances of the group analysis and on the DC we performed. Our study chose GB and CCC algorithms for their implementation readiness inside the MIP platform. However, other promising or most common classifiers such as Random Forest (67), Support Vector Machine, or semi-supervised heuristic approaches (68) should be tested further on. The development of new feature selection strategies to identify relevant and nonredundant feature subsets, innovative ensemble, and deep learning algorithms for clinical classification (69) should be explored too. Except for the MMSE score, in the “single-case analysis” part, aggregated outcomes coming from neuropsychological tests were acquired and dichotomized into normal/abnormal according to different local clinical practices and protocols. The same caveat applies to CSF biomarkers. We cannot claim that different tests and protocols may have different impacts on DC. Furthermore, we included only one biomarker of brain amyloidosis (i.e., CSF AB1-42) without considering the amyloid-PET imaging data. We chose not to include amyloid-PET because this examination could not be processed by the MIP because of the lack of an *ad hoc* automatic pipeline of imaging analysis. Indeed, it will be important to integrate this type of pipeline so that future studies will also take advantage of multimodal MRI and PET imaging information. Lastly, the limited sample size in the single-case analysis of MCI patients warrants replication in larger studies.

## Conclusion

Thanks to the MIP platform, we are assisting to a radical change in neuroscience that is symbolized by moving away from traditional syndromic diagnosis toward diagnoses based on biological signatures, while not ruling out the importance of clinician supervision. Advanced statistics, notably data mining and ML tools, are the armamentarium for this paradigm shift. The novel approach we demonstrated does not constitute a substitute for the classical hypothesis-led approach conducted in neuroscience so far. Rather, it is a complementary methodology that allows a better understanding of the complexity of the brain and its diseases. The present study also demonstrated an effect on DC and an influence on the physicians' clinical thinking and decision making when the MIP outcomes were added to other biomarkers.

The disease signature, emerging from big-data analyses and innovative ML approaches, seems to play a promising role in patients' stratification within future clinical trials or observational studies. This methodology may help to identify new targets for intervention, guide better care, and lead to precision diagnostics.

## Data Availability Statement

Publicly available datasets were analyzed in this study. This data can be found here: https://www.neugrid2.eu/index.php/data-portfolio/; http://adni.loni.usc.edu/data-samples/access-data/; https://services.humanbrainproject.eu/oidc/login.

## Ethics Statement

The patients/participants provided their written informed consent to participate in this study.

## Author Contributions

AR: conceptualization, methodology, software, formal analysis, investigation, writing, and visualization. SD: methodology, software, formal analysis, investigation, resources, data curation, and writing. FP: software and formal analysis. SG: methodology and validation. CM: resources and data curation. GC and GS: software. PT and GB: validation. AN: software and resources. MB: resources. MR: methodology. SF: project administration. CG: writing. FT: supervision. GF: resources and supervision. PR: software and supervision. J-FD: writing and supervision. FK: software, data curation, and supervision. SC: methodology, validation, writing, and supervision. ED'A: conceptualization, software, investigation, resources, writing - review - editing, and supervision. All authors contributed to the article and approved the submitted version.

## Conflict of Interest

The authors declare that the research was conducted in the absence of any commercial or financial relationships that could be construed as a potential conflict of interest.
