# Supplementary material for: Medical Informatics Platform (MIP): A Pilot Study Across Clinical Italian Cohorts
Source: Front Neurol. 2020 Sep 23;11:1021. doi: 10.3389/fneur.2020.01021 (PMC7538836; doi:10.3389/fneur.2020.01021)
Supplement: Supplementary file 1 [file Table_1.docx]

| **Inclusion/Exclusion Criteria** | | | |
| --- | --- | --- | --- |
|  | **CN** | **MCI** | **AD** |
| **ADNI** | - No Memory Complaint.  - Mini-Mental State Exam (MMSE) score between 24 and 30 (inclusive).  - Clinical Dementia Rating (CDR) = 0.  - Cognitively normal, based on an absence of significant impairment in cognitive functions or activities of daily living.  - Normal memory function documented by scoring at specific cutoffs on the Logical Memory II subscale (delayed Paragraph Recall) from the Wechsler Memory Scaled - Revised (the maximum score is 25):  a) greater than or equal to 9 for 16 or more years of education;  b) greater than or equal to 5 for 8-15 years of education;  c) greater than or equal to 3 for 0-7 years of education. | - Memory complaint by subject or study partner that is verified by a study partner.  - MMSE score between 24 and 30.  - CDR = 0.5.  - General cognition and functional performance sufficiently preserved such that a diagnosis of AD cannot be made by the site physician at the time of the screening visit.  - Abnormal memory function documented by scoring below the education adjusted cutoff on the Logical Memory II subscale (Delayed Paragraph Recall) from the Wechsler Memory Scale –Revised (the maximum score is 25):  a) less than or equal to 8 for 16 or more years of education;  b) less than or equal to 4 for 8-15 years of education;  c) less than or equal to 2 for 0-7 years of education. | - Memory complaint by subject or study partner that is verified by a study partner.  - MMSE between 20 and 26 (inclusive).  - CDR = 0.5, 1.0.  - National Institute of Neurological and Communicative Disorders and Stroke and the Alzheimer's Disease and Related Disorders Association (NINCDS-ADRDA) criteria for probable AD.  - Abnormal memory function documented by scoring below the education adjusted cutoff on the Logical Memory II subscale (Delayed Paragraph Recall) from the Wechsler Memory Scale – Revised (the maximum score is 25):  a) less than or equal to 8 for 16 or more years of education;  b) less than or equal to 4 for 8-15 years of education;  c) less than or equal to 2 for 0-7 years of education. |
| **EDSD** | CN were required to be free of cognitive complaints and to have performed according to the age and education adjusted norms in all subtests of the Consortium to Establish a Registry of Alzheimer's Disease (CERAD) testing battery (Morris et al., 1989). | MCI were diagnosed according to the Petersen criteria, exhibiting subjective and objective cognitive impairment (exceeding − 1.5 standard deviations in the Consortium to Establish a Registry of Alzheimer's Disease (CERAD) testing battery, controlled for age and education) and being free of dementia (Petersen, 2004). | Dementia patients were diagnosed with clinically probable AD according to the NINCDS-ADRDA criteria (McKhann et al., 1984) and were required to be free of any other significant neurological, psychiatric, or medical conditions. |
| **FBF** | CN subjects were enrolled in the study if: (i) their brain scan was judged normal on visual assessment by the neuroradiologist in charge; (ii) cognitive impairment was not found on neuropsychological testing (a posteriori exclusion criteria).  The neuropsychological tests investigated: verbal (Bab-cock, Rey Auditory Verbal Learning Test) and non-verbal (Rey figure recall) long-term learning, verbal fluency (letter and category), psychomotor speed (Trial Making A) and visuo-spatial abilities (Rey figure copy). Exclusion criteria were the presence of clinical, psychiatric, neurological, or neuropsychological impairment, and of cerebrovascular disease or positive finding at neuroradiological evaluation of the MR. | Subjects were included as single or multi-domain MCI according to National Institute on Aging and Alzheimer's Association (NIA-AA) criteria (Albert et al., 2011). | Subjects were included as AD according to core clinical criteria for probable AD (McKhann et al., 2011). Exclusion criteria were: (i) evidence of depression or dysthymia; (ii) other major systemic, psychiatric or neurological illnesses; (iii) other causes of focal or diffuse brain damage on their MRI scan (e.g., lacunae and extensive cerebrovascular disorders). |
| **Besta** | Subjects were included as CN if cognitively unimpaired (equivalent scores ≥ 1 on neuropsychological tests). | Subjects were included as single or multi-domain MCI according to NIA-AA criteria (Albert et al., 2011). | Subjects were included as (probable) amnestic or non-amnestic AD according to NIA-AA criteria (McKhann et al., 2011). |
| **CHT Niguarda** | Subjects cognitively unimpaired (MMSE: ≥ 24, and equivalent scores ≥ 1 on neuropsychological tests exploring the principal cognitive domains). | Subjects were included as MCI following criteria according to:  [Early detection of Alzheimer's disease: new diagnostic criteria](https://www.ncbi.nlm.nih.gov/pmc/articles/PMC3181912/)  (Dubois et al., 2009). | Subjects were included as (probable) amnestic or non-amnestic AD according to NIA-AA criteria (McKhann et al., 2011). |
| **Piramal** | NA | MCI (amnestic or non-amnestic/single or multiple domain) or mild dementia possibly due to AD (Albert et al., 2011):  - Age: 55 – 90 years.  - Education: at least 5 years.  - Modified Hachinski Ischemic Scale: score <4.  - Available informant / caregiver  - Written informant consent  - Exposure to amyloid targeting agents.  - Use of the following drugs in the last 4 weeks:  (i) narcotic analgesics and high dose antidepressants  (ii) high dose typical and atypical neuroleptics  (iii) drugs with significant central nervous system anticholinergic activity  -Pregnancy or breast feeding  -Neurological or psychiatric illness precluding enrollment.  -Visual or auditory acuity inadequate for neuropsychological tests.  -Enrolment in studies not compatible with PET scanning. | NA |

*Table Sup 1 reports eligibility criteria for Research data sets (ADNI, EDSD), Clinical data sets (FBF, Besta, CHT-Niguarda), and Piramal data set. Acronyms: CN: Normal Cognition; MCI: Mild Cognitive Impairment; AD: Alzheimer’s Dementia; NA: Not Applicable.*
