# Supplementary material for: Medical Informatics Platform (MIP): A Pilot Study Across Clinical Italian Cohorts
Source: Front Neurol. 2020 Sep 23;11:1021. doi: 10.3389/fneur.2020.01021 (PMC7538836; doi:10.3389/fneur.2020.01021)
Supplement: Supplementary file 4 [file Table_4.docx]

| *BEFORE DISCLOSING MIP INFORMATION* | | | | | |
| --- | --- | --- | --- | --- | --- |
|  |  | **Rater 1** | **Rater 2** | **Rater 3** | **Rater 4** |
| **PATHOPHYSIOLOGICALLY AD RELATED** | MCI due to AD | 11 | 12 | 7 | 11 |
| **NOT PATHOPHYISIOLOGICALLY AD RELATED** | MCI not due to AD | 20 | 26 | 23 | 15 |
|  | MCI due to FTD | 2 | 0 | 6 | 2 |
|  | MCI due to VD | 0 | 0 | 1 | 1 |
|  | MCI due to Psychiatric disorders | 0 | 0 | 1 | 0 |
|  | SNAP | 0 | 0 | 0 | 3 |
|  | Normal Aging | 5 | 0 | 0 | 6 |

| *AFTER DISCLOSING MIP INFORMATION* | | | | | |
| --- | --- | --- | --- | --- | --- |
|  |  | **Rater 1** | **Rater 2** | **Rater 3** | **Rater 4** |
| **PATHOPHYSIOLOGICALLY AD RELATED** | MCI due to AD | 10 | 12 | 7 | 11 |
| **NOT PATHOPHYSIOLOGICALLY AD RELATED** | MCI not due to AD | 21 | 26 | 22 | 15 |
|  | MCI due to FTD | 2 | 0 | 7 | 2 |
|  | MCI due to VD | 0 | 0 | 1 | 1 |
|  | MCI due to Psychiatric disorders | 0 | 0 | 1 | 0 |
|  | SNAP | 0 | 0 | 0 | 3 |
|  | Normal Aging | 5 | 0 | 0 | 6 |

*Table Sup 4 reports the diagnostic categories expressed by the four physicians for the 38 Piramal subjects before and after disclosure of the MIP information.*
