# Supplementary material for: Medical Informatics Platform (MIP): A Pilot Study Across Clinical Italian Cohorts
Source: Front Neurol. 2020 Sep 23;11:1021. doi: 10.3389/fneur.2020.01021 (PMC7538836; doi:10.3389/fneur.2020.01021)
Supplement: Supplementary file 5 [file Table_5.docx]

| **Data sets**  **Comparison** | **AD** | **MCI** | **CN** |
| --- | --- | --- | --- |
|  | **p-values** | | |
|  |  |  |  |
| FBF vs Besta | 0.519 | 0.439 | 0.052 |
| FBF vs CHT Niguarda | NA | 0.052 | 0.412 |
| Besta vs CHT Niguarda | NA | 0.081 | 0.130 |
| Research data sets (EDSD vs ADNI) | 0.777 | 0.557 | 0.668 |
| Clinical data sets (FBF vs Besta vs CHT Niguarda) | NA | 0.066 | 0.093 |
| Clinical data sets vs EDSD | 0.091 | 0.064 | 0.062 |
| Clinical data sets vs ADNI | 0.243 | 0.118 | 0.663 |
| Clinical data sets vs Research data sets | 0.056 | 0.052 | 0.052 |
|  |  |  |  |

*Table Sup 5 shows p-values from the MANOVA test after removal of subjects due to MRI artifacts, prevention of class-imbalance, and maximization of clinical data sets’ representativeness. 788 subjects were discarded to preserve the 3 diagnostic classes in the Italian centres (i.e.: FBF, Besta, CHT Niguarda). It was not possible to preserve AD of CHT Niguarda because only one subject had a good 3DT1 MRI, and this class was discarded. All of the p-values were greater than 0.05, which shows the comparability between the diagnostic classes of the 5 data sets. Acronyms: Clinical data sets: FBF+Besta+CHT Niguarda; Research data sets: EDSD+ADNI; NA: impossibility to run MANOVA due to diagnostic class missing.*
