# Supplementary material for: Medical Informatics Platform (MIP): A Pilot Study Across Clinical Italian Cohorts
Source: Front Neurol. 2020 Sep 23;11:1021. doi: 10.3389/fneur.2020.01021 (PMC7538836; doi:10.3389/fneur.2020.01021)
Supplement: Supplementary file 6 [file Table_6.docx]

| **Feature_ID** | **Feature_Name** | **Weghted_Importance** |
| --- | --- | --- |
| PB125 | ab1_42 | 0.132 |
| PB126 | t_tau | 0.112 |
| PB127 | p_tau | 0.093 |
| PB122 | Minimentalstate | 0.072 |
| CM2 | lefthippocampus | 0.017 |
| CM4 | leftamygdala | 0.012 |
| CM6 | X_4thventricle | 0.012 |
| PB101 | rightscasubcallosalarea | 0.011 |
| PB52 | leftioginferioroccipitalgyrus | 0.009 |
| PB95 | rightppplanumpolare | 0.009 |
| PB130 | educationyears | 0.008 |
| PB46 | leftfrpfrontalpole | 0.008 |
| CM12 | Leftpcggposteriorcingulategyrus | 0.008 |
| CM3 | rightamygdala | 0.008 |
| CM8 | Leftmcggmiddlecingulategyrus | 0.008 |
| CM7 | Rightmcggmiddlecingulategyrus | 0.008 |
| PB119 | rightttgtransversetemporalgyrus | 0.007 |
| PB16 | leftpallidum | 0.007 |
| PB39 | rightcuncuneus | 0.007 |
| PB4 | rightcaudate | 0.007 |
| PB40 | leftcuncuneus | 0.007 |
| PB5 | leftcaudate | 0.007 |
| PB74 | leftmtgmiddletemporalgyrus | 0.007 |
| PB107 | rightsmgsupramarginalgyrus | 0.006 |
| PB113 | rightstgsuperiortemporalgyrus | 0.006 |
| PB118 | lefttrifgtriangularpartoftheinferiorfrontalgyrus | 0.006 |
| PB120 | leftttgtransversetemporalgyrus | 0.006 |
| PB27 | leftbasalforebrain | 0.006 |
| PB42 | leftententorhinalarea | 0.006 |
| PB54 | leftitginferiortemporalgyrus | 0.006 |
| PB56 | leftliglingualgyrus | 0.006 |
| PB71 | rightmsfgsuperiorfrontalgyrusmedialsegment | 0.006 |
| PB86 | leftphgparahippocampalgyrus | 0.006 |
| PB91 | rightpogpostcentralgyrus | 0.006 |
| PB94 | leftporgposteriororbitalgyrus | 0.006 |
| CM9 | rightacgganteriorcingulategyrus | 0.006 |
| PB100 | leftptplanumtemporale | 0.005 |
| PB112 | leftsplsuperiorparietallobule | 0.005 |
| PB128 | profession | 0.005 |
| PB13 | rightinflatvent | 0.005 |
| PB24 | cerebellarvermallobulesiv | 0.005 |
| PB41 | rightententorhinalarea | 0.005 |
| PB51 | rightioginferioroccipitalgyrus | 0.005 |
| PB55 | rightliglingualgyrus | 0.005 |
| PB7 | leftcerebellumexterior | 0.005 |
| PB8 | rightcerebellumwhitematter | 0.005 |
| PB80 | leftopifgopercularpartoftheinferiorfrontalgyrus | 0.005 |
| PB83 | rightpcuprecuneus | 0.005 |
| PB9 | leftcerebellumwhitematter | 0.005 |
| PB98 | leftprgprecentralgyrus | 0.005 |
| CM10 | Leftacgganteriorcingulategyrus | 0.005 |
| CM5 | X_3rdventricle | 0.005 |
| PB1 | rightaccumbensarea | 0.004 |
| PB105 | rightsmcsupplementarymotorcortex | 0.004 |
| PB109 | rightsogsuperioroccipitalgyrus | 0.004 |
| PB11 | leftcerebralwhitematter | 0.004 |
| PB114 | leftstgsuperiortemporalgyrus | 0.004 |
| PB123 | Rightlateralventricle | 0.004 |
| PB124 | leftlateralventricle | 0.004 |
| PB14 | leftinflatvent | 0.004 |
| PB15 | rightpallidum | 0.004 |
| PB2 | leftaccumbensarea | 0.004 |
| PB20 | leftthalamusproper | 0.004 |
| PB26 | cerebellarvermallobulesviiix | 0.004 |
| PB28 | rightbasalforebrain | 0.004 |
| PB3 | brainstem | 0.004 |
| PB34 | leftangangulargyrus | 0.004 |
| PB35 | rightcalccalcarinecortex | 0.004 |
| PB36 | leftcalccalcarinecortex | 0.004 |
| PB43 | rightfofrontaloperculum | 0.004 |
| PB45 | rightfrpfrontalpole | 0.004 |
| PB53 | rightitginferiortemporalgyrus | 0.004 |
| PB67 | rightmpogpostcentralgyrusmedialsegment | 0.004 |
| PB73 | rightmtgmiddletemporalgyrus | 0.004 |
| PB75 | rightocpoccipitalpole | 0.004 |
| PB84 | leftpcuprecuneus | 0.004 |
| PB87 | rightpinsposteriorinsula | 0.004 |
| PB92 | leftpogpostcentralgyrus | 0.004 |
| PB97 | rightprgprecentralgyrus | 0.004 |
| PB99 | rightptplanumtemporale | 0.004 |
| CM11 | Rightpcggposteriorcingulategyrus | 0.004 |
| PB10 | rightcerebralwhitematter | 0.003 |
| PB102 | leftscasubcallosalarea | 0.003 |
| PB106 | leftsmcsupplementarymotorcortex | 0.003 |
| PB108 | leftsmgsupramarginalgyrus | 0.003 |
| PB110 | leftsogsuperioroccipitalgyrus | 0.003 |
| PB111 | rightsplsuperiorparietallobule | 0.003 |
| PB115 | righttmptemporalpole | 0.003 |
| PB116 | lefttmptemporalpole | 0.003 |
| PB117 | righttrifgtriangularpartoftheinferiorfrontalgyrus | 0.003 |
| PB12 | csfglobal | 0.003 |
| PB17 | rightputamen | 0.003 |
| PB23 | opticchiasm | 0.003 |
| PB25 | cerebellarvermallobulesvivii | 0.003 |
| PB32 | leftaorganteriororbitalgyrus | 0.003 |
| PB44 | leftfofrontaloperculum | 0.003 |
| PB47 | rightfugfusiformgyrus | 0.003 |
| PB48 | leftfugfusiformgyrus | 0.003 |
| PB50 | leftgregyrusrectus | 0.003 |
| PB59 | rightmfcmedialfrontalcortex | 0.003 |
| PB60 | leftmfcmedialfrontalcortex | 0.003 |
| PB64 | leftmogmiddleoccipitalgyrus | 0.003 |
| PB68 | leftmpogpostcentralgyrusmedialsegment | 0.003 |
| PB69 | rightmprgprecentralgyrusmedialsegment | 0.003 |
| PB72 | leftmsfgsuperiorfrontalgyrusmedialsegment | 0.003 |
| PB76 | leftocpoccipitalpole | 0.003 |
| PB78 | leftofugoccipitalfusiformgyrus | 0.003 |
| PB82 | leftorifgorbitalpartoftheinferiorfrontalgyrus | 0.003 |
| PB85 | rightphgparahippocampalgyrus | 0.003 |
| PB88 | leftpinsposteriorinsula | 0.003 |
| PB90 | leftpoparietaloperculum | 0.003 |
| PB93 | rightporgposteriororbitalgyrus | 0.003 |
| PB96 | leftppplanumpolare | 0.003 |
| PB103 | rightsfgsuperiorfrontalgyrus | 0.002 |
| PB121 | apoe4 | 0.002 |
| PB18 | leftputamen | 0.002 |
| PB19 | rightthalamusproper | 0.002 |
| PB21 | rightventraldc | 0.002 |
| PB22 | leftventraldc | 0.002 |
| PB30 | leftainsanteriorinsula | 0.002 |
| PB31 | rightaorganteriororbitalgyrus | 0.002 |
| PB33 | rightangangulargyrus | 0.002 |
| PB37 | rightcocentraloperculum | 0.002 |
| PB38 | leftcocentraloperculum | 0.002 |
| PB49 | rightgregyrusrectus | 0.002 |
| PB58 | leftlorglateralorbitalgyrus | 0.002 |
| PB6 | rightcerebellumexterior | 0.002 |
| PB61 | rightmfgmiddlefrontalgyrus | 0.002 |
| PB62 | leftmfgmiddlefrontalgyrus | 0.002 |
| PB63 | rightmogmiddleoccipitalgyrus | 0.002 |
| PB65 | rightmorgmedialorbitalgyrus | 0.002 |
| PB66 | leftmorgmedialorbitalgyrus | 0.002 |
| PB70 | leftmprgprecentralgyrusmedialsegment | 0.002 |
| PB77 | rightofugoccipitalfusiformgyrus | 0.002 |
| PB79 | rightopifgopercularpartoftheinferiorfrontalgyrus | 0.002 |
| PB81 | rightorifgorbitalpartoftheinferiorfrontalgyrus | 0.002 |
| PB89 | rightpoparietaloperculum | 0.002 |
| CM1 | righthippocampus | 0.002 |
| PB104 | leftsfgsuperiorfrontalgyrus | 0.001 |
| PB129 | familiarity | 0.001 |
| PB29 | rightainsanteriorinsula | 0.001 |
| PB57 | rightlorglateralorbitalgyrus | 0.001 |
| PB131 | gender | 0 |

*Table Sup 6 reports the ranking of the features used by Gradient Boosting (GB) to discriminate NC, MCI, and AD subjects. For each feature, “weight importance” is reported.*
