# Supplementary material for: Medical Informatics Platform (MIP): A Pilot Study Across Clinical Italian Cohorts
Source: Front Neurol. 2020 Sep 23;11:1021. doi: 10.3389/fneur.2020.01021 (PMC7538836; doi:10.3389/fneur.2020.01021)
Supplement: Supplementary file 7 [file Image_1.pdf]

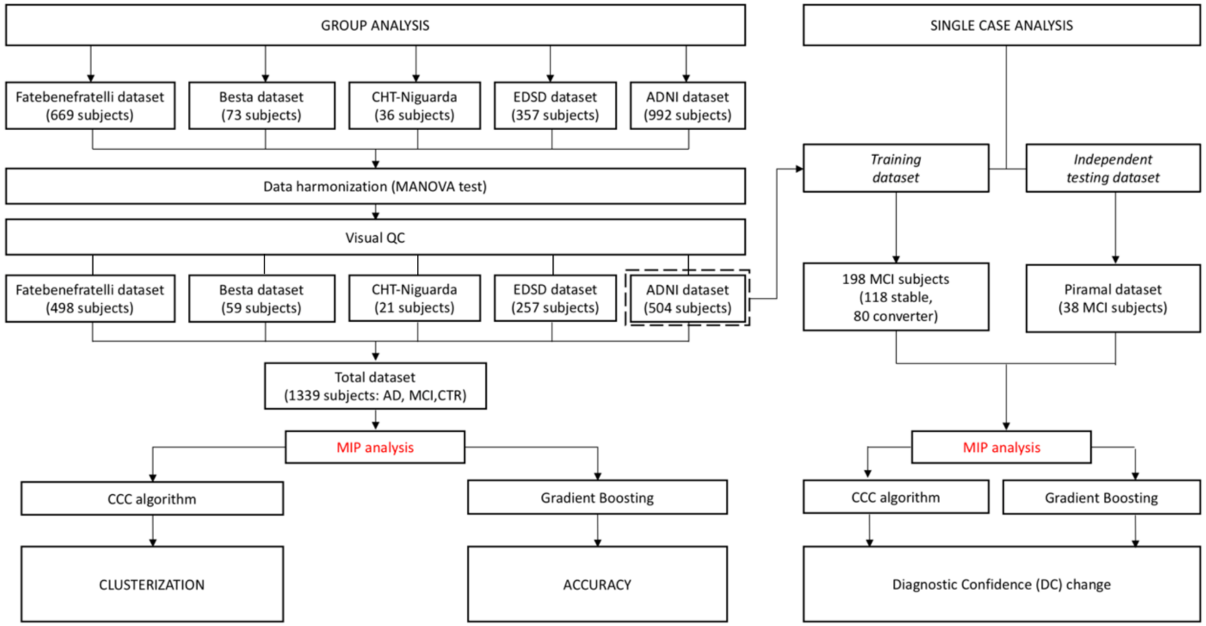

Figure Sup 1 shows the study workflow. The study was organized in two parts: (1) "Group analysis" where we selected 1339 subjects from clinical data sets (i.e., Fatebenefratelli, Besta, CHT-Niguarda) and research data sets (i.e., EDSD, ADNI) and (2) "Single case analysis" where we analysed 38 MCI Piramal subjects as independent validation data cohort. In the "Single case analysis" 198 MCI subjects of the ADNI with 2 years of follow up were used as training data set.

Data harmonization was performed in order to combine data from all of the different cohorts. Expert evaluators performed visual quality control (QC) on the automated ROI segmented with the Neuromorphometric pipeline on T13D scans.

In the group analysis, the MIP tools (i.e., GB and CCC) were trained and tested using nested 10-fold cross validation and hold-out validation strategies. GB and CCC were used to investigate the MIP performances in classifying patients as CN, MCI, and AD and find new potential homogeneous clusters, respectively.

In the single case analysis, GB and CCC were tested on 38 well-characterized MCI Piramal cases to investigate the perception of the diagnostic utility of the MIP tools by four expert physicians at three leading Italian dementia centres (IRCCS Fatebenefratelli, IRCCS Carlo Besta, CHT-Niguarda hospital).

Acronyms: MIP: Medical Informatics Platform; CN: Cognitive Normal; MCI: Mild Cognitive Impairment; AD: Alzheimer's dementia; QC: Quality Control; DC: Diagnostic Confidence.
