# Supplementary material for: Medical Informatics Platform (MIP): A Pilot Study Across Clinical Italian Cohorts
Source: Front Neurol. 2020 Sep 23;11:1021. doi: 10.3389/fneur.2020.01021 (PMC7538836; doi:10.3389/fneur.2020.01021)
Supplement: Supplementary file 8 [file Image_2.pdf]

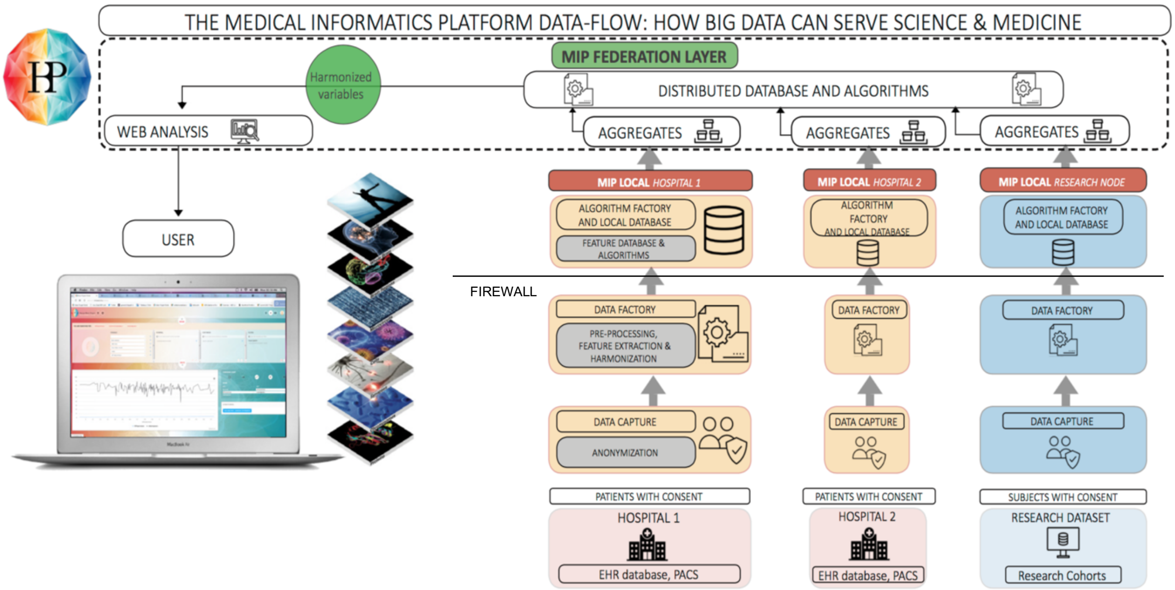

Figure Sup 2 shows the Medical Informatics Platform (MIP) structure. MIP allows physicians and neuroscientists to run analyses on health records distributed across different hospitals and research centres without moving the data outside their original storage environment. MIP provides 2 data sources, i.e., research data (EDSD, ADNI) and clinical data coming from integrated hospitals (Fatebenefratelli, Besta, CHT-Niguarda, etc..). All of the data are (pseudo-)anonymized and matched to the MIP Common Data Element (CDE) schema where more than 200 variables (for the complete list see: http://www.centroalzheimer.it/public/CDE_MIP.xls) were collected for all the data cohorts. Thanks to the "data-factory" layer, the data are processed with ad hoc pipelines (e.g., Neuromorphometrics or others) and the features extracted from T13D scans combined with neurophysiological tests, -omics, and medical records. MIP has a user-friendly graphical user interface (GUI) that is accessible from a web-browser (https://services.humanbrainproject.eu/oidc/login). Every user must obtain an HBP Identity Account to login into the platform. The GUI allows the running of statistical analyses, data-mining tools, machine learning algorithms, and predictive models (i.e., algorithm factory layer). The MIP federation layer, thanks to a declarative SQL language extended with parallelisms, allows to query and aggregate the results from the distributed nodes. Therefore, coming from the MIP web-portal, all of the queries and analyses first pass through the federation layer. The queries are forwarded to each local database that accesses the hospital data or to the local "algorithm-factory" for results computations. The aggregated data and results are sent back to the federation layer, which collects and merges all the information from the different hospitals. Because only aggregated and alphanumeric averaged results leave hospitals, the data and patients' privacy are fully preserved.
