# Supplementary material for: Medical Informatics Platform (MIP): A Pilot Study Across Clinical Italian Cohorts
Source: Front Neurol. 2020 Sep 23;11:1021. doi: 10.3389/fneur.2020.01021 (PMC7538836; doi:10.3389/fneur.2020.01021)
Supplement: Supplementary file 9 [file Image_3.pdf]

1) Left hippocampus

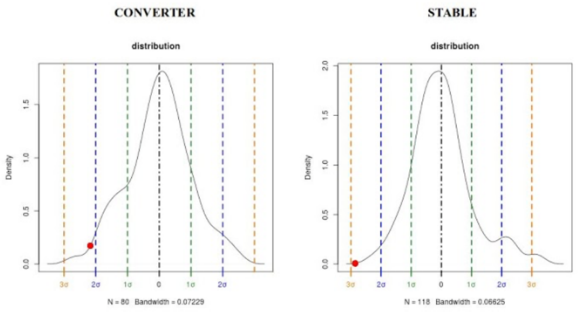

2) Right hippocampus

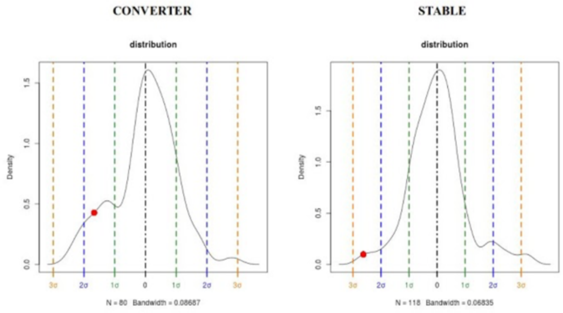

3) Left middle cingulate gyrus

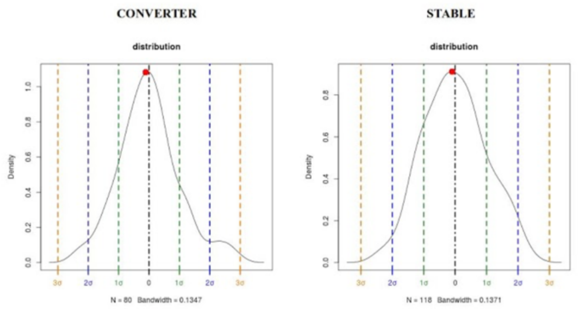

4) Left anterior cingulate gyrus

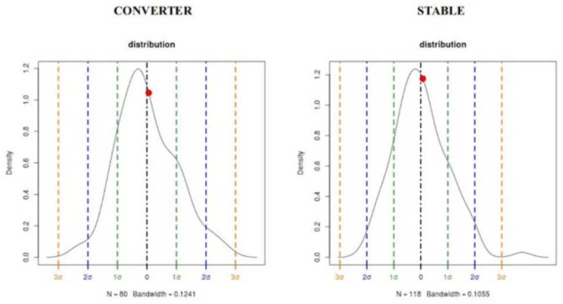

5) Left posterior cingulate gyrus

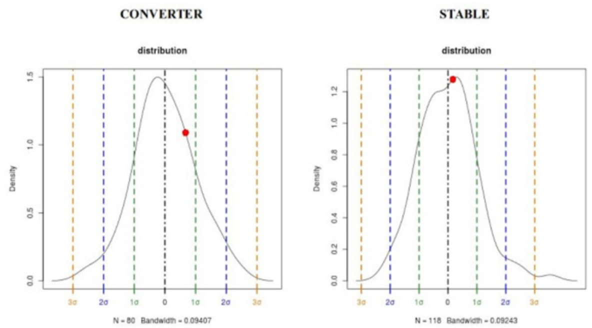

6) Right entorhinal volume

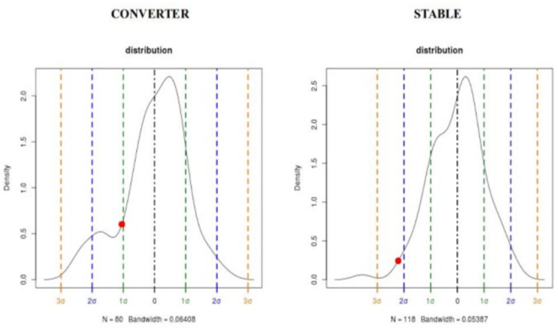

7) Left entorhinal volume

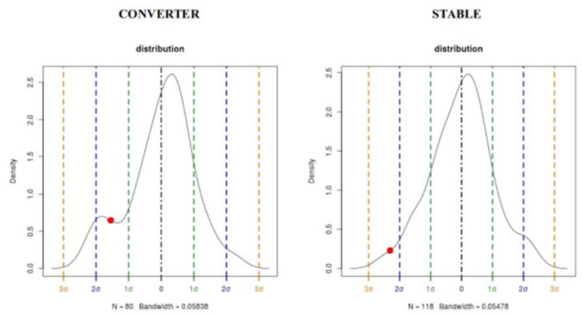

8) Right middle temporal gyrus

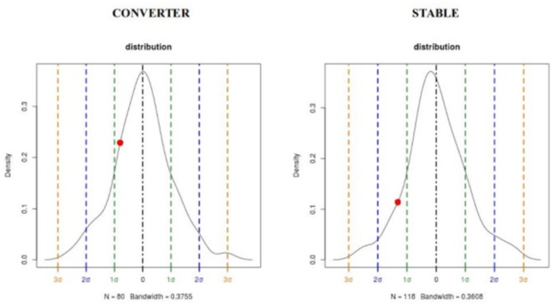

9) Right parahippocampal gyrus

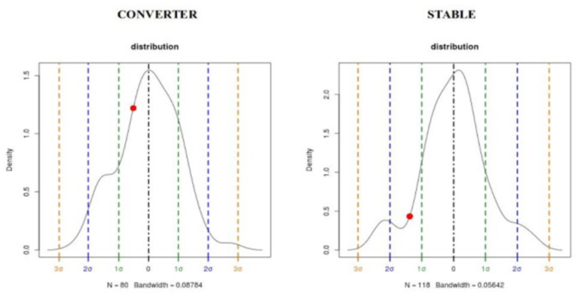

10) Right superior temporal gyrus

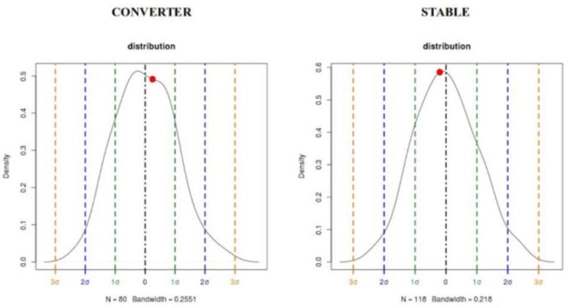

Mean (0) ----- *1 SD (1σ)* ----- *2 SD (2σ)* ----- *3 SD (3σ)*

Figure Sup 3 shows an example of the density function distributions and standard deviations from 10 imaging biomarkers that were quantified using the MIP Neuromorphometrics pipeline. The volumetric distribution on the left was derived from MCI CONVERTER and the distribution on the right was derived from MCI STABLE from ADNI data set. Subject value is plotted as a red dot on the density function.
