# Supplementary material for: Medical Informatics Platform (MIP): A Pilot Study Across Clinical Italian Cohorts
Source: Front Neurol. 2020 Sep 23;11:1021. doi: 10.3389/fneur.2020.01021 (PMC7538836; doi:10.3389/fneur.2020.01021)
Supplement: Supplementary file 10 [file Image_4.pdf]

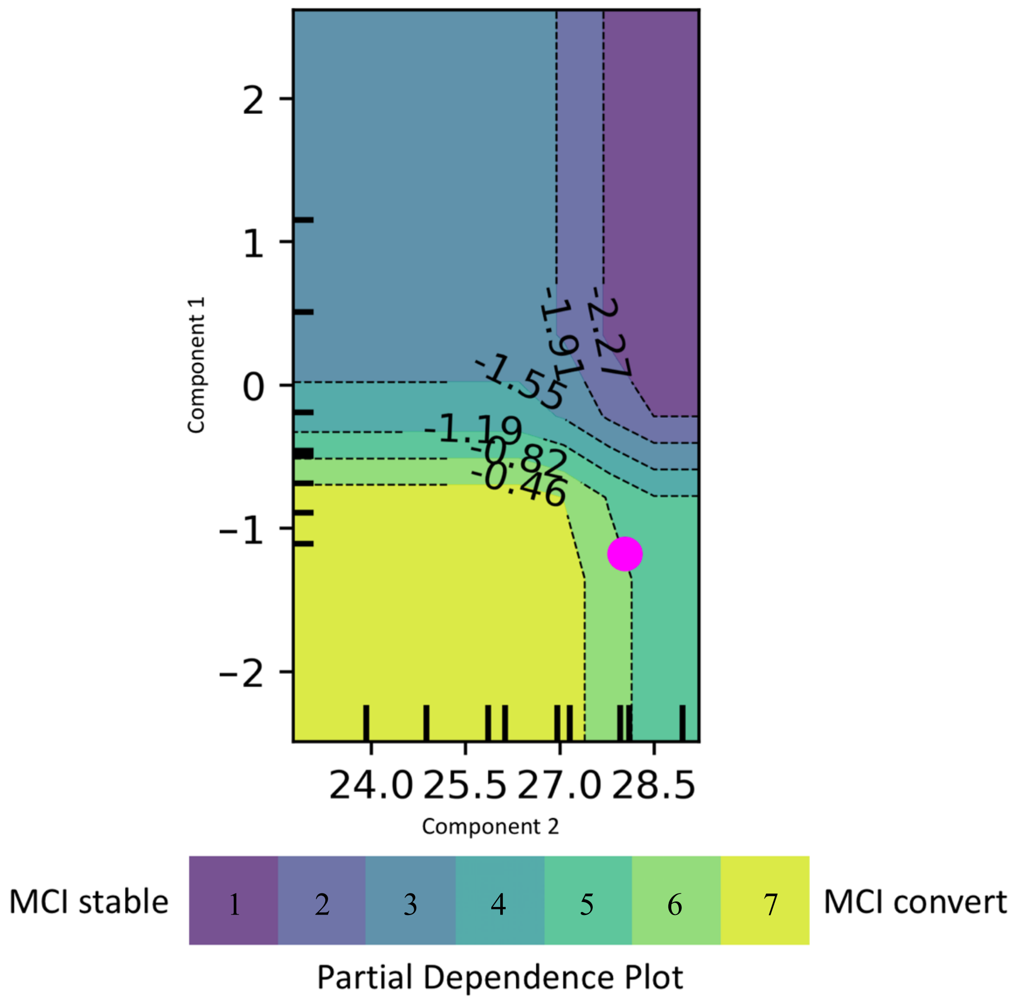

Figure Sup 4 shows an example of the Partial Dependence Plot (PDP) generated by the Gradient Boosting classifier. There are 7 stages of probability. Cooler colours represent maximum probability of belonging to the MCI stable state. Brighter colours represent maximum probability of belonging to the MCI converter to AD. Magenta point represents where the Piramal subject falls in the 2D probabilistic space, which was trained on 198 ADNI subjects.
